# Development of High-Performance Coconut Oil-Based Rigid Polyurethane-Urea Foam: A Novel Sequential Amidation and Prepolymerization Process

**DOI:** 10.1021/acsomega.3c09598

**Published:** 2024-03-07

**Authors:** Louell Nikki A. Hipulan, Roger G. Dingcong, Dave Joseph E. Estrada, Gerard G. Dumancas, John Christian S. Bondaug, Arnold C. Alguno, Hernando P. Bacosa, Roberto M. Malaluan, Arnold A. Lubguban

**Affiliations:** † Center for Sustainable Polymers, Mindanao State University − Iligan Institute of Technology, A. Bonifacio Avenue, Iligan9200, Philippines; ‡ Environmental Science Graduate Program, Department of Biological Sciences, Mindanao State University − Iligan Institute of Technology, A. Bonifacio Avenue, Iligan 9200, Philippines; § Chemical Engineering Program, College of Technology, University of San Agustin, General Luna St., Iloilo 5000, Philippines; ∥ Department of Chemistry, The University of Scranton, Scranton, Pennsylvania 18510, United States; ⊥ Department of Physics, Mindanao State University − Iligan Institute of Technology, A. Bonifacio Avenue, Iligan 9200, Philippines; # Department of Chemical Engineering and Technology, Mindanao State University − Iligan Institute of Technology, A. Bonifacio Avenue, Iligan 9200, Philippines

## Abstract

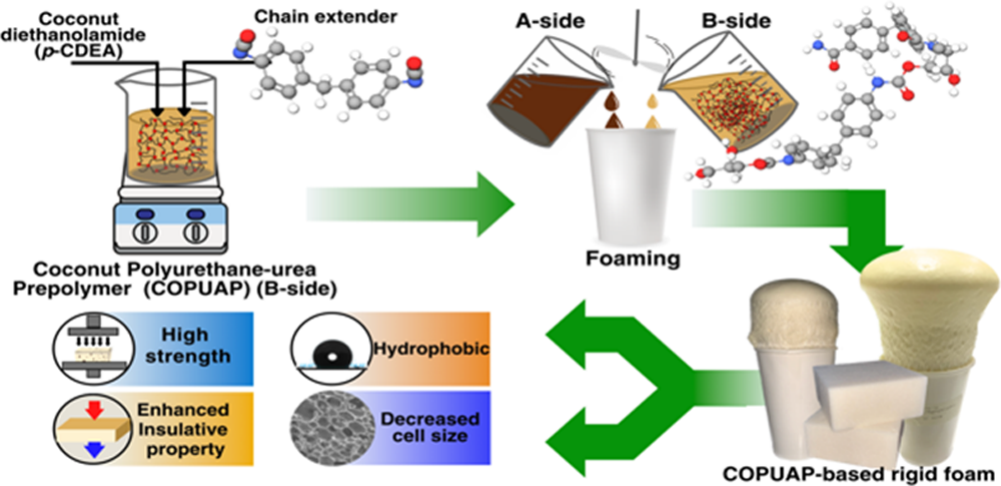

The utilization of coconut diethanolamide (*p*-CDEA) as a substitute polyol for petroleum-based polyol in fully biobased rigid polyurethane-urea foam (RPUAF) faces challenges due to its short chain and limited cross-linking capability. This leads to compromised cell wall resistance during foam expansion, resulting in significant ruptured cells and adverse effects on mechanical and thermal properties. To address this, a novel sequential amidation-prepolymerization route was employed on coconut oil, yielding a hydroxyl-terminated poly(urethane-urea) prepolymer polyol (COPUAP). Compared to *p*-CDEA, COPUAP exhibited a decreased hydroxyl value (496.3–473.2 mg KOH/g), an increase in amine value (13.464–24.561 mg KOH/g), and an increase in viscosity (472.4–755.8 mPa·s), indicating enhanced functionality of 34.3 mgKOH/g and chain lengthening. Further, COPUAP was utilized as the sole B-side polyol in the production of RPUAF (PU-COPUAP). The improved functionality of COPUAP and its improved cross-linking capability during foaming have significantly improved cell morphology, resulting in a remarkable 4.7-fold increase in compressive strength (132–628 kPa), a 3.5-fold increase in flexural strength (232–828 kPa), and improved insulation properties with a notable decrease in thermal conductivity (48.02–34.52 mW/m·K) compared to PU-CDEA in the literature. Additionally, PU-COPUAP exhibited a 16.5% increase in the water contact angle (114.93° to 133.87°), attributing to the formation of hydrophobic biuret segments and a tightly packed, highly cross-linked structure inhibiting water penetration. This innovative approach sets a new benchmark for fully biobased rigid foam production, delivering high load-bearing capacity, exceptional insulation, and significantly improved hydrophobicity.

## Introduction

Polyurethane (PU) materials are widely known for their diverse range of properties and have been used as elastomers, fibers, foams, and surface coatings in a wide array of settings.^1−4^ Rigid PU foams as a specific type of PU have been extensively used in modern building insulation, refrigerated storage, and construction due to their excellent thermal insulation, high strength-to-weight ratio, and low absorption properties.^5,6^ The versatility of rigid PU foam materials largely depends on the reaction of isocyanate and petroleum-derived polyols forming urethane linkages,^7,8^ but with pressing environmental concerns associated with petroleum-based products, there has been a growing emphasis on developing alternative biobased polyols.^9−11^

Vegetable oils derived from rapeseed,^11^ castors,^12^ and soybean^13^ have been investigated as promising sources of biobased polyols for PU production. Chemical modification of the carbon chains in the fatty acids of these oils can result in high hydroxyl functionality polyols^12,13^ which can provide multiple reactive groups for isocyanate cross-linking, thereby enhancing the mechanical and thermal properties of the final product.^9,12^

Among the vegetable oils explored for biobased polyols in PU production, castor oil stands out for its extensive study in the rigid polyurethane industry owing to its high functionality.^12^ However, despite its promising attributes, its continued reliance on polyol replacements has opened the door to alternative solutions.^12,14,15^ Recent research, including the work by Lee et al. and Hejna et al., has revealed that complete replacement of conventional polyols with castor oil-based counterparts results in inferior mechanical properties compared to formulations with partial replacements.^12,14,15^ This limitation has prompted the exploration of other biobased polyols, such as coconut oil.

The potential of coconut (*Cocos nucifera*) oil (CO) as a sustainable alternative to castor oil has garnered attention due to its abundance, especially in Asia and Africa.^16^ According to the United States Department of Agriculture Production Supply and Distribution, CO ranks as the seventh most abundant vegetable oil in the world, compared to castor, linseed, and corn oil, which notably fails to achieve a prominent ranking in global abundance.^17^ This comparison indicates the importance of coconut oil, highlighting its role as a valuable resource material with the capacity to support various applications, such as polyurethane formulations. Moreover, this recognition lays the groundwork for future research into utilizing coconut oil derivatives, such as coconut fatty acid distillate (CFAD),^18^ coconut monoglyceride (CMG),^19^ or cooking oil wastes,^20^ highlighting the importance of exploring coconut oil-based polyols for the production of high-quality rigid foams.

The exploration of coconut oil’s potential in sustainable rigid polyurethane foam development began with pioneering work by Khanmohammadi et al. They elucidated a chemical transformation process involving coconut oil fatty acids, diethanolamine, and glycerol, leading to significant advancements in material synthesis.^21^ This process culminated in the synthesis of *p*-CDEA, which is a highly functional compound. Paruzel et al. synthesized rigid polyurethane (PU) foam utilizing coconut oil alongside polycarbonate and polyurethane scraps.^6^ Subsequent studies by Leng et al. and Dingcong et al. further extended this research, focusing on the utilization of *p*-CDEA in enhancing the mechanical and thermal properties of rigid PU foam. These collective studies contribute to the evolving understanding of coconut oil-derived compounds in the context of polymeric materials.^16,22,23^

The recent studies mentioned above have demonstrated that *p*-CDEA when used as a polyol component for rigid polyurethane-urea foam (RPUAF) application yielded poor mechanical and thermal properties compared to petroleum-based PU foam.^16,23^ This is due to the short to medium chain length of *p*-CDEA that affects the cross-linking density during foaming, which leads to weaker cell walls.^8,16^ The presence of amino esters initiates a catalytic effect and leads to a rapid bubble formation. Also, the presence of short-chain molecules such as free glycerol in *p*-CDEA decelerates polymer chain propagation. This phenomenon ultimately reduces the resistance of cell walls during foam cell expansion.^16,23^ As a result, the *p*-CDEA-based RPUAF has inferior mechanical and thermal properties compared to the traditional petroleum-based rigid PU foam.^16,17,24^ Thus, it is important to enhance significant factors that affect RPUAF’s structural integrity, which include the polyol’s chemical properties and cross-linking capacity.^25,26^

Prepolymerization, which has been used in the field of PU production, has been employed as a viable solution in this study to overcome the short-chain drawback of using *p*-CDEA in rigid foam production.^16,23^ Prepolymerization is a reaction mechanism in the production of PU that serves to cross-link soft and hard segments as well as provide control in viscosity, molecular weight, and reactivity.^26,27^ By facilitating polyol chain lengthening and cross-linking capability, the formation of uniform and enhanced cellular structure can prevent foam rupture during cell growth, thereby improving the mechanical and thermal properties of the foam.

Recent studies have primarily focused on the prepolymerization of petroleum-based polyols for use in PU film coating.^27,28^ Additionally, the majority of prepolymerization research related to biobased polyols utilizes isocyanate-terminated urethane prepolymerization for the PU film.^26,29,30^ As for hydroxyl-terminated prepolymerization, Badri and his colleagues synthesized an OH-terminated prepolymer for PU coating using sorbitol-based palm kernel oil polyol (SBPKO).^27^

*p*-CDEA-based RPUAF would be more competitive and appealing for application in the biobased PU foam industry if it could demonstrate comparable performance in mechanical and thermal properties relative to commercially available rigid PU foams in the market. A fully biobased RPUAF which is produced without any need for petroleum-based polyol substitution is highly desirable in the pursuit of sustainable development goals. Therefore, the study on the effect of the prepolymerization step on *p*-CDEA and the effect of the resulting CO poly(urethane-urea) prepolymer (COPUAP) polyol on RPUAF’s mechanical and thermal performance is highly necessitated.

**Figure 1:**
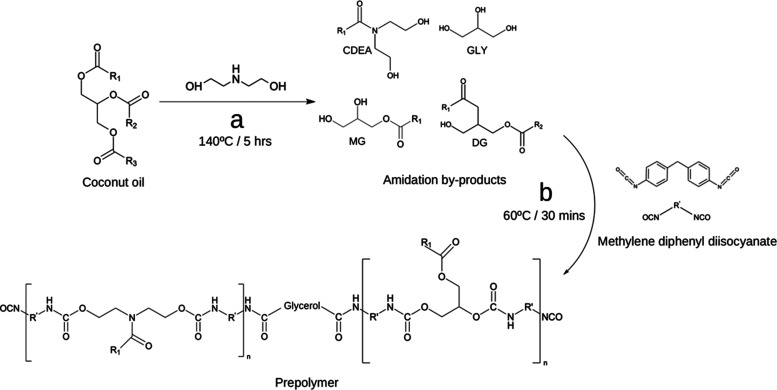
Synthetic route of (a) coconut diethanolamide (*p*-CDEA) via direct amidation of coconut oil; (b) coconut oil-based hydroxyl-terminated poly(urethane-urea) prepolymer polyol (COPUAP).

In this study, a novel sequential amidation-prepolymerization process was used to enhance the functionality and chain lengthening properties of the COPUAP polyol. Purification-free *p*-CDEA was subjected to hydroxyl-terminated prepolymerization with the addition of methylene diphenyl diisocyanate (MDI) (Figure 1a). During prepolymerization, the amino esters, particularly the secondary amine-bearing component, react with diisocyanate forming urea linkages.^23^ The subsequent formation of urethane-urea linkages during prepolymerization leads to chain lengthening and an increase in functionality, which produces COPUAP (Figure 1b).^31^ The viscosity of *p*-CDEA and COPUAP was used as an indirect indicator to investigate the occurrence of chain lengthening, while other methods such as Fourier transform infrared (FTIR) spectroscopy and amine value (NHV) and hydroxyl value (OHV) determination were employed to provide information on the changes in functional groups associated with chain lengthening of COPUAP. Further, COPUAP was used as the sole B-side polyol component in the production of RPUAF to determine whether there was an improvement in its mechanical and thermal properties. The physical and mechanical properties were studied via density, compressive, and flexural strength measurements. The morphological property of RPUAFs was characterized using scanning electron microscopy (SEM). Thermal characteristics were analyzed through differential scanning calorimetry (DSC), thermogravimetric analysis (TGA), and heat flow meter (HFM) test. Surface hydrophobicity was evaluated using water contact angle measurement.

The purpose of this study is to produce a fully biobased RPUAF, utilizing COPUAP as the main B-side component resulting in a cellular foam structure improvement with enhanced mechanical, thermal, and hydrophobic properties. This study is the first to explore the use of a sequential amidation-prepolymerization method using CO to produce a polyol with improved functionality and a relatively longer chain length.

## Materials and Methods

### Materials

CO was procured from a local supplier (Iligan City, Philippines). Reagent-grade diethanolamine [NH_2_(C_2_H_4_OH)_2_] (DEA) was provided by Ajax Finechem. Polymeric methylene diphenyl diisocyanate (MDI) (PAPI 27 from Dow Chemical, with NCO functionality and content of 2.7 and 31.4 wt %, respectively), zinc oxide (ZnO), silicone surfactant (Dabco DC 2585), catalyst (Polycat 8) from Air Products and Chemicals, Inc., and petroleum-based polyether polyol (Voranol 490 from Dow Chemical, has an alcohol functionality of 4.3, an average molecular weight of 460, and a hydroxyl number of 490) were provided by Chemrez Technologies, Inc., Philippines. Methyl red was obtained from Sigma-Aldrich. All chemicals were utilized as received without any additional purification.

### Synthesis of Coconut Oil Polyurethane-Urea Prepolymer Polyol

The COPUAP was prepared via the reaction of synthesized *p*-CDEA with 12% MDI loading. The chain-extension reaction was carried out in a beaker equipped with a magnetic stirrer and an alcohol thermometer (Figure S1). The *p*-CDEA was heated below 60 °C under agitation while gradually adding MDI.^30^ After 30 min,^32,33^ the COPUAP was cooled down at ambient temperature, and its viscosity was evaluated using a DV3T viscometer (AMETEK Brookfield, Middleborough, MA) at 25 ± 0.5 °C. The attainment of the torque ranging from 30 to 40% was indicative of the completion of the prepolymerization process.^23^

### Preparation of Rigid Foam

A modified rigid PU foam formulation described by Dingcong et al. was used for foam formulation in this study.^23^Table 1 outlines the formulation of RPUAF, which were denoted as PU-V490, PU-CDEA, and PU-COPUAP for petroleum-based (VORANOL 490), *p*-CDEA-based, and COPUAP-based, respectively. RPUAF was prepared by using high-speed stirring. The B-side components of RPUAF consist of polyol, catalyst, and surfactant, while the A-side is the polymeric MDI. The foams were obtained by speed-mixing the B-side components in a 500 mL paper cup, degassing for 10 s, and mixing in the A-side component thoroughly at 3000–4000 rpm. After being foamed, the samples were cured for 7 days to ensure that the chemical reaction between isocyanate and polyol was complete.

**Table 1:** Formulations of Rigid Poly(urethane-urea) Foam (RPUAF) at Different Polyol Substitutions: Coconut Diethanolamide (*p*-CDEA), Coconut Oil Polyurethane-Urea Prepolymer (COPUAP), and VORANOL 490

concentration, pphp^a^				
foam formulation	components	PU-V490	PU-CDEA	PU-COPUAP
B-side materials				
polyol	V490	100	0	0
	*p*-CDEA	0	100	0
	COPUAP	0	0	100
catalyst	Polycat 8	1.75	1.75	1.75
surfactant	Dabco 2585	3	0.5	0.5
blowing agent	water	2	2	2
A-side materials				
isocyanate^b^	MDI PAPI 27	144.94	119.259	118.435

a Concentrations of each ingredient are expressed in parts per hundred parts (pphp) of polyol, adhering to the convention where the cumulative total of all polyols amounts to 100 parts.

b The isocyanate index denotes the ratio of the utilized isocyanate quantity to the theoretically required amount, multiplied by 100.

### Biobased Polyol Characterization

The amine (NHV) and hydroxyl (OHV) values were determined using ASTM D2074-07 and ASTM D4274-99 Test Method D, respectively.^12^ The viscosity of the polyols was digitally obtained using a DV3T (AMETEK Brookfield, Middleborough, MA) rotary viscometer with an adapter, temperature probe, and temperature control unit at 25.0 ± 0.5 °C, maintaining a torque ranging from 15 to 30% for COPUAP and 30 to 40% for *p*-CDEA.^23^ The FTIR spectra for both *p*-CDEA and COPUAP were validated by using an FTIR spectrophotometer (IRTracer-100, Shimadzu Corp., Kyoto, Japan). At a resolution of 2 cm^–1^, a total of 40 scans from each sample were collected at wavelengths ranging from 4000 to 500 cm^–1^.

### Rigid Polyurethane-Urea Foam Characterization

The temperature profile of foaming was obtained using a thermocouple, which was carefully inserted into the central part of RPUAF. The temperature and time of foam expansion were recorded.^36^^,^^37^ A cup test method used was modified from ASTM-7487 to determine gel time. After curing, the foam was cut into various sizes by using an electric cutting machine to prepare it for subsequent characterization. The density and percent closed-cell content of both COPUAP and *p*-CDEA-based RPUAFs were determined using a Quantachrome Ultrapyc 1200e Automatic Gas Pycnometer 02112-1 by ASTM 2856-94 (Quantachrome Instruments Corp., Boynton Beach, Florida).^34^

The foaming ratio can be calculated based on the density change due to prepolymerization, as expressed by eqn 1 where ρ_o_ is the density of the polymer before foaming and ρ_p_ is the density of the microcellular foamed plastic produced after foaming.^35^ Therefore, this foaming ratio can be considered to represent the number of cells inside the microcellular foamed plastic.
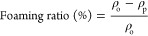
1

The morphological properties of the surfaces of the foam were analyzed through scanning electron microscopy/energy dispersive X-ray spectroscopy (SEM/EDX) using a JEOL JSM-6510LA SEM instrument (JEOL, Ltd., Tokyo, Japan). The samples were sputter coated with 10 nm of platinum before image capturing using SEM. The cell morphology was captured using JEOL scanning electron microscope software (JEOL, Japan), and the cell size distribution was carefully analyzed using ImageJ software (NIH, US). The average diameter of the cells in the micrographs, *D*, was calculated using eqn 2.^34^
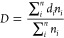
2where *n*_*i*_ is the number of cells in the captured SEM with a perimeter-equivalent diameter of *d*_*i*_ and *I* is the total number of pores that is greater than 200 ensuring that it exceeds 200 to guarantee the accuracy of the average pore size measurement.

### Swelling and Gel Content Test

The swelling and gel content test was determined using the swelling procedure of Pongmuksuwan and Harnnarongchai.^36^ First, the cut samples (5 mm × 10 mm × 2 mm) were weighed (*W*_0_) and then immersed in 200 mL of toluene at 25 °C for 48 h to swell. After, the swollen RPUAF was weighed (*W*_1_) after removing the excess toluene on the surface using tissue. The sorption of the RPUAFs was calculated using eqn 3
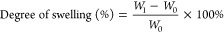
3

Gel content was then determined after the swelling study. The swollen samples were dried at 25 °C for 24 h, and the dried sample was weighed (*W*_2_). The gel content was calculated by using eq 4.
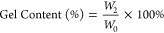
4

The volume fraction (*V*_p_) and degree of swelling (DS) of the cross-linked PU were determined via eqs 5 and 6
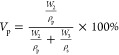
5where ρ_p_ is the density of polyurethane (g/cm^3^), *W*_3_ is the weight of solvent (g), and ρ_s_ is the density of the solvent (g/cm^3^).
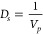
6

The cross-linking density (*v*_e_, mol/cm^3^) was determined using the swelling method according to the Flory–Rehner equation, as shown in eq 6.
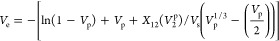
7where *V*_s_ is the molar volume of the toluene (106.3 cm^3^/mol),^41^*V*_p_ is the volume fraction of swollen polyurethane, and *X*_12_ is the Flory–Huggins polymer–solvent interaction parameter (0.45 for the polyurethane in toluene).^36^

### Mechanical Test

To determine the compressive and flexural strength of the rigid foams, a universal testing machine (Shimadzu Autograph AGS-X Series, Shimadzu Corp., Kyoto, Japan) was used by ASTM D1621-04a and ISO 178:2010, respectively. The Trapezium X material testing software (Shimadzu Corp., Kyoto, Japan) was utilized to calculate and display the results.^38,39^ For the compressive strength test, the foams were prepared by cutting them into 25.4 ± 0.2 mm thickness and 50.00 ± 0.2 mm in length and width and then measuring them at 25 °C. In the three-point flexural test, foam sticks measuring 200.0 ± 0.2 mm in height, 25.0 ± 0.2 mm in length, and 12.5 ± 0.2 mm in width were used. These foam sticks were placed on two solid supports, which were spaced 65 mm apart.^40^

### Thermal Characterization

The thermal conductivity for both RPUAF was tested using a FOX 200 heat flow meter (Laser-Comp, Wakefield, MA) according to ASTM C518-2017 (sample size 150 × 150 × 20 mm).^39^ The thermal performance of the prepared foams was evaluated on a PerkinElmer DSC 4000 (PerkinElmer, Waltham, MA) with a heating rate of 10 °C/min (sample weight 10–20 mg), and the thermal stability of both foams was measured with TGA performed under nitrogen atmosphere at 10 °C/min from −50 to 800 °C using a Shimadzu DTG 60H (Shimadzu Corp., Kyoto, Japan).^23^

### Contact Angle and Hydrophobicity

The measurement of the water contact angle was conducted using a Theta Lite (Biolin Scientific, Espoo, Finland). The foam samples were cut into small pieces and placed on glass slides. A droplet of deionized water, 4 μL in volume, was placed on the foam surface through a microsyringe. One Attension software (Nanoscience Instruments, Inc., Phoenix, AZ) was used to determine the contact angle.

## Results and Discussion

### Effect of Prepolymerization on the Properties of Coconut Oil-Based Polyol

Recent studies have demonstrated the potential of *p*-CDEA as a polyol component in the production of rigid RPUAF. However, its inferior mechanical and thermal properties compared to commercially available petroleum-based PU foam have been attributed to its short chain length, resulting in reduced cell wall resistance strength during foaming.^16,23^ The presence of amino esters containing secondary amine, which is in equilibrium with diethanolamide, induces an autocatalytic effect that accelerates the reaction between water and isocyanate, leading to bubble size expansion. The short-chain molecules such as free glycerol in *p*-CDEA decelerate polymer chain propagation ultimately compromising the foam’s cellular morphology, which directly affects its mechanical and thermal properties.^16,23^ To overcome this challenge, prepolymerization is employed to enhance the short-chain disadvantage associated with the use of *p*-CDEA as a polyol component by facilitating chain lengthening and functionality enhancement. A good cellular wall structure is crucial for preventing foam rupture during cell growth, thereby improving the mechanical and thermal properties of the foam.^33,34^

The resulting COPUAP at varying MDI loading was subjected to NHV and OHV determination to provide insights into the functionality of the prepolymer. Generally, a higher amine and hydroxyl value of polyol indicates a higher number of reactive groups per unit mass.^38^ This higher functionality can lead to increased cross-linking density in the final product.^39^

**Figure 2:**
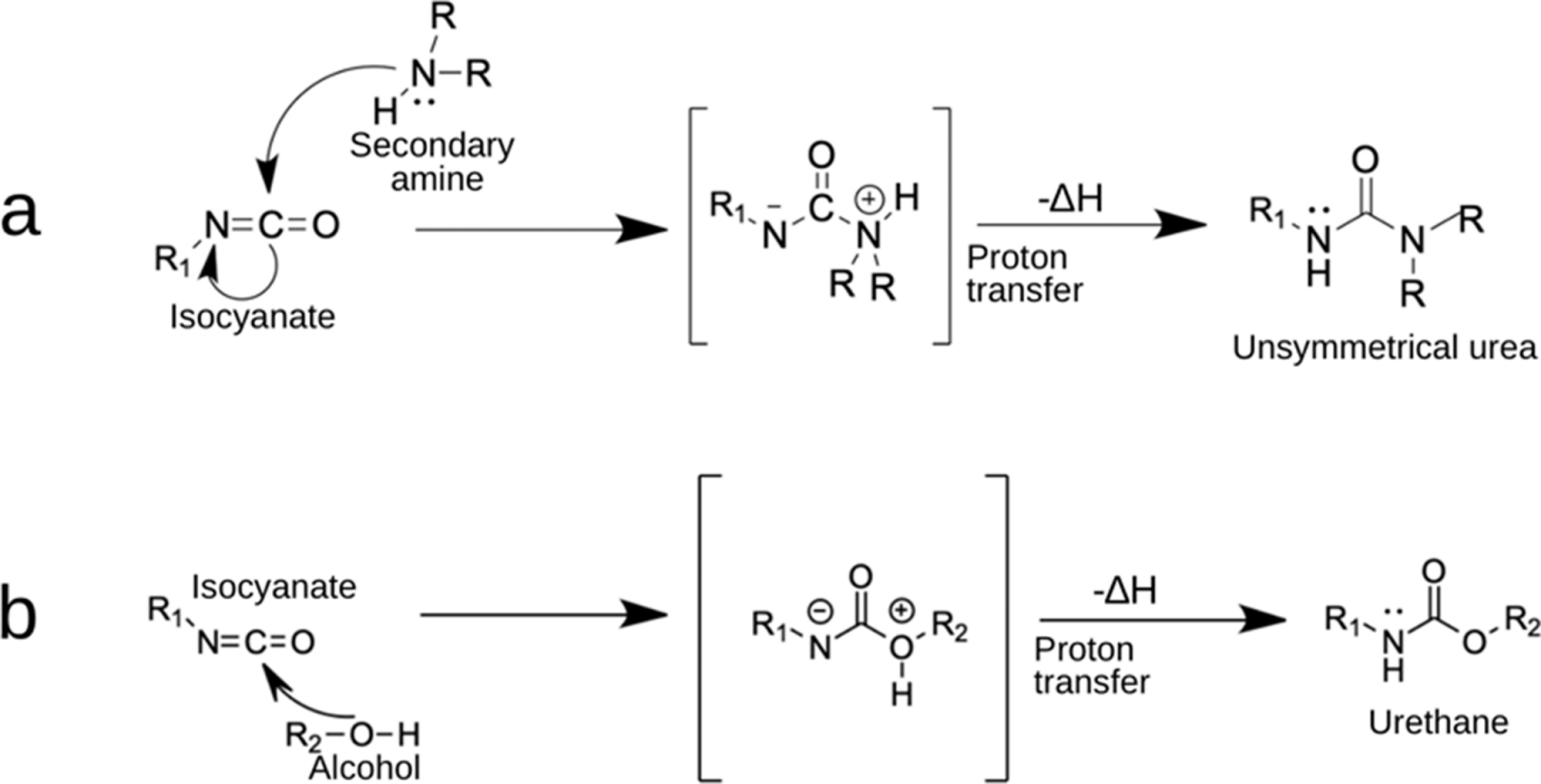
Chemical reaction of (a) urea and (b) urethane formation during prepolymerization of coconut diethanolamide (*p*-CDEA).

As previously reported, *p*-CDEA contains amino esters that feature reactive secondary amine groups, and coexisting secondary amine groups act as a chain-extender.^23^ They can undergo a reaction with isocyanate during prepolymerization, consequently leading to the formation of urea linkages, as depicted in Figure 2a.^40^ Another reaction that occurs during prepolymerization is the reaction of the hydroxyl group in *p*-CDEA with isocyanate to form urethane functional groups (Figure 2b).^42^ The chemical reactions depicted in Figure 2 result from a nucleophilic attack of the amine and hydroxyl group in the chain extender to the carbon in the isocyanate group leading to the formation of urea and urethane linkages, respectively, and lengthening of the prepolymer chain.^29^

**Figure 3:**
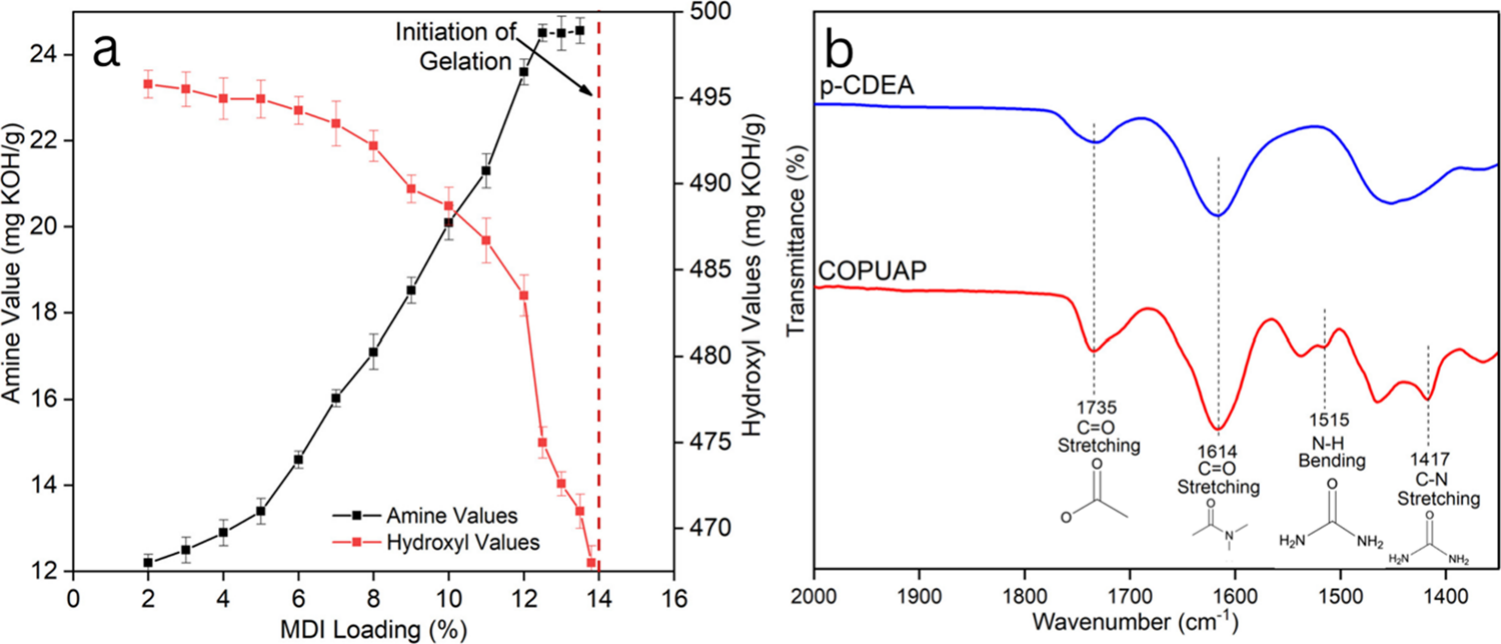
(a) Amine and hydroxyl values of coconut oil polyurethane-urea prepolymer (COPUAP) as a function of methylene diphenyl diisocyanate (MDI) loading; (b) Fourier transform infrared (FTIR) spectra for both biobased polyols: COPUAP and coconut diethanolamide (*p*-CDEA) at bands of 2000–1300 cm^–1^.

The chain lengthening during prepolymerization results in an increase of amine groups due to the formation of urethane or urea linkages. These reactions lead to the incorporation of additional amine groups into the prepolymer chain, thus increasing its amine value and enhancing its functionality.^31,33,43^ It is evident in Figure 3a that there is a significant 82.4% increase in NHV at 12% MDI loading prepolymerization, which implies an increase of reactive sites per molecule of COPUAP.

During prepolymerization, urethane linkages form through the reaction of an isocyanate group and a hydroxyl group in *p*-CDEA, as depicted in Figure 2b. This reaction leads to a reduction in the OHV. As evidenced by the results presented in Figure 3a, the OHV decreased by 4.6% after the prepolymerization of *p*-CDEA at 12% MDI loading. To further investigate the formation of chain propagation and functionality enhancement during prepolymerization, *p*-CDEA and COPUAP were characterized using FTIR spectroscopy over the wavenumber of 4000–500 cm^–1^. The most important characteristic bands are located at 2000–1300 cm^–1^ since C=N stretching vibrations of amines and C=O of carbonyl stretching are usually associated with this range. Both polyols’ FTIR spectra in Figure 3b indicate an amide carbonyl band found at 1614 cm^–1^, which confirms that the process of amidation has successfully taken place, thereby resulting in the presence of diethanolamide.^20^ In the spectra of COPUAP, characteristic peaks were observed at 1536 and 1515 cm^–1^, which are attributed to the bending vibration of the urea-based N–H group formed by the reaction of −NH and −NCO during prepolymerization.^26^ Additionally, the observed peak at 1417 cm^–1^ of COPUAP is due to the stretching vibration of the urea-based C–N group formed by the reaction of −NH and −NCO during prepolymerization.^26^ Furthermore, the stretching vibration of the C=O carbonyl bond in urethane appears at 1735 cm^–1^ for COPUAP polyol.^42^

**Figure 4:**
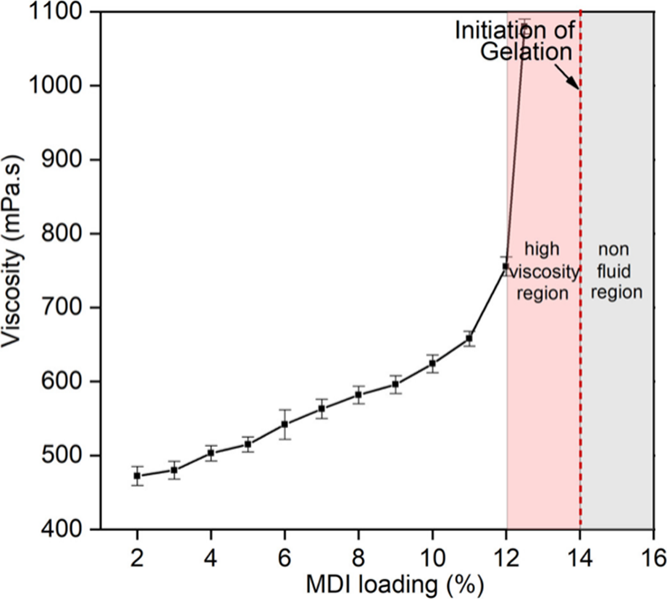
Viscosity of coconut oil polyurethane-urea prepolymer polyol (COPUAP) with increasing methylene diphenyl diisocyanate (MDI) loading (%) measured at a constant temperature of 25 ± 0.5 °C.

The lengthening of the PU-urea prepolymer chain increases the viscosity of COPUAP polyol (Figure S2).^30,44^ As the chain grows longer, the viscosity of the system increases due to the lengthening of the polymer chains.^16^Figure 4 highlights the effect of the prepolymerization process on the viscosity of COPUAP, demonstrating a notable increase in viscosity with increasing MDI loading. However, when the MDI loading is greater than 12%, the blend homogeneity is compromised, resulting in very high viscosity, rendering the resulting prepolymer unsuitable for foam production. Therefore, the optimal loading for the COPUAP chain lengthening formulation was chosen to be at 12% MDI loading.

### Preparation and Characterization of Poly(urethane-urea) Rigid Foams

**Figure 5:**
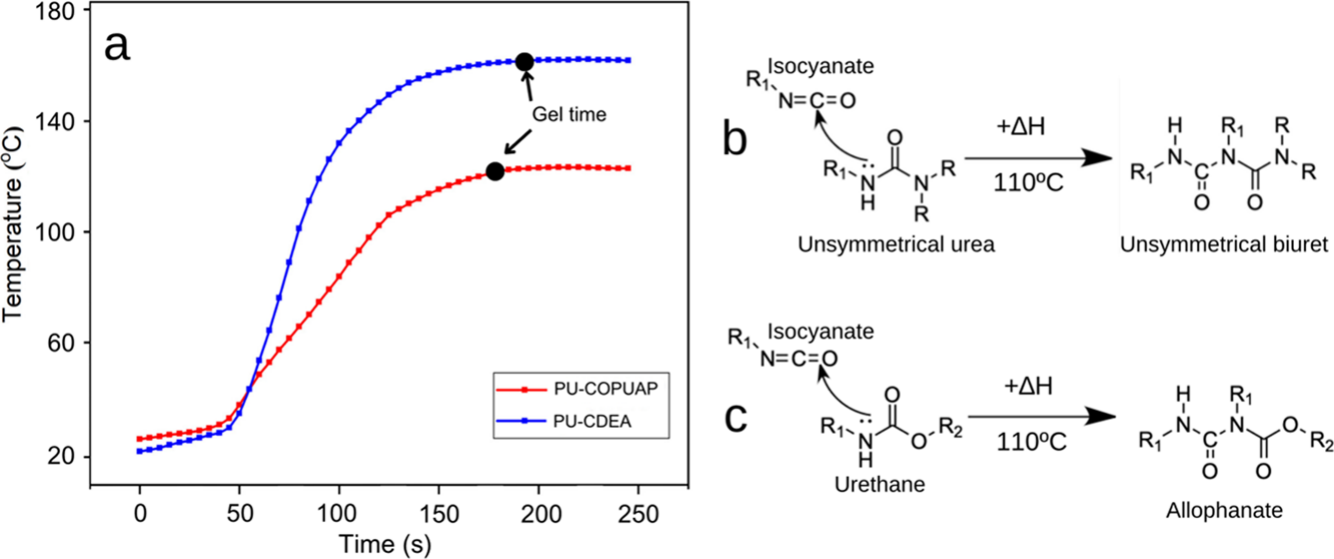
(a) Temperature profile of coconut diethanolamide (*p*-CDEA) and coconut oil polyurethane-urea prepolymer (COPUAP) during foaming with gel time; chemical reaction of (b) catalytic nucleophilic addition–elimination to produce biuret and (c) allophanate bond during foam formation.

RPUAF was synthesized using the formulations specified in Table 1. The temperature profile presented in Figure 5a revealed that the PU-CDEA foam formation proceeded through a more exothermic reaction compared to PU-COPUAP. The lower temperature profile of the PU-COPUAP formation can be attributed to the endothermic formation of biuret, a urea-derived functionality (Figure 5b), and allophanate, a urethane-derived functionality (Figure 5c), which are prominent during the foaming process of PU-COPUAP.^33^ The formation of biuret and allophanate is a trimerization reaction, which is an endothermic process that necessitates the use of heat (of about 110 °C) to activate the reaction.^39^ As a result, the energy absorption during the formation of biuret and allophanate influences the temperature profile of the foaming process of PU-COPUAP by limiting the temperature increase during foaming. Moreover, the reaction of the PU-CDEA formation exhibits an exothermic nature. This can be attributed to the reaction of secondary amine groups and isocyanate, leading to the formation of urea linkages as depicted in Figure 2a. Such reaction is the dominant reaction in PU-CDEA formation, especially during the initial stages of foam rise.^33^ Urea formation in the PU-CDEA foaming process has a high heat of formation, which can affect the temperature profile of PU-CDEA.

The formation of biuret and allophanate can lead to cross-linking during foam formation.^45^ Allophanate is an intermediate compound that is formed from the reaction between isocyanate and urethane groups present in the foam-forming system, whereas biuret is formed from urea and isocyanate groups. These reactions result in the formation of cross-links between polymer chains in the foam. Moreover, cross-linking can affect the gel time during foaming.^46^ Gel time (the time when the mixture changed from viscous to gel) was studied since it indicates the beginning of polymerization.^45^ The formation of chemical bonds between the polymer chains reduces the mobility of the polymer molecules and increases their cross-linking.^39^ As shown in Figure 5a, there was a decrease in gel time of PU-COPUAP in comparison to PU-CDEA. Moreover, the high rate of reaction can affect the degree of cross-linking and the final morphology of the foam; it can lead to issues, such as uneven cell sizes and density variations, which negatively impact the foam’s mechanical and physical properties.^47^

The experimentally obtained foaming ratio is shown in Table 2. In Table 2, it can be seen that the foaming ratio decreased by 3% subsequent to the incorporation of the prepolymerization process. This decrease can be attributed to compromised mixing efficacy due to the increase of viscosity; consequently, this results to uneven expansion and a lower foaming ratio.

**Table 2:** Foaming Ratio of Coconut Diethanolamide-Based (PU-CDEA) and Coconut Oil Polyurethane-Urea Prepolymer (PU-COPUAP) Rigid Foam

foam sample	polymer density before foaming	polymer density after foaming	foaming ratio, %
PU-CDEA	1108	57.1	94.85
PU-COPUAP	1122	93.5	91.67

Table 3 illustrates the cross-linking density (*v*_e_), degree of swelling, and gel content of both PU-CDEA and PU-COPUAP. With prepolymerization, the cross-link density increases, while the degree of swelling decreases. The *v*_*e*_ of polyurethanes within the range 10^–3^–10^–4^ mol/cm^3^ have been extensively reported,^41^ whereas the *v*_e_ of the polyurethanes in this study are approximately 4 × 10^–8^ to 7 × 10^–8^ mol/cm^3^, which are significantly lower than those reported.^41,53,54^ Materials with low *v*_e_ exhibit higher energy dissipation than those exhibited with higher *v*_e_. PU-CDEA has a gel content of 89.49%, which increases up to 91.55% with prepolymerization. The increment of gel content and cross-linking density values can be attributed to the formation of biuret and allophanate linkages between the molecules, which participate in cross-linking.

**Table 3:** Crosslinking Density, Swelling Ratio, Gel Content of Coconut Diethanolamide-Based (PU-CDEA) and Coconut Oil Polyurethane-Urea Prepolymer (PU-COPUAP) Rigid Foam

foam sample	cross-linking density × 10^–8^ (mol/m^3^)	degree of swelling (%)	gel content (%)
PU-CDEA	3.57	260.58	89.49
PU-COPUAP	6.86	133.86	91.55

**Figure 6:**
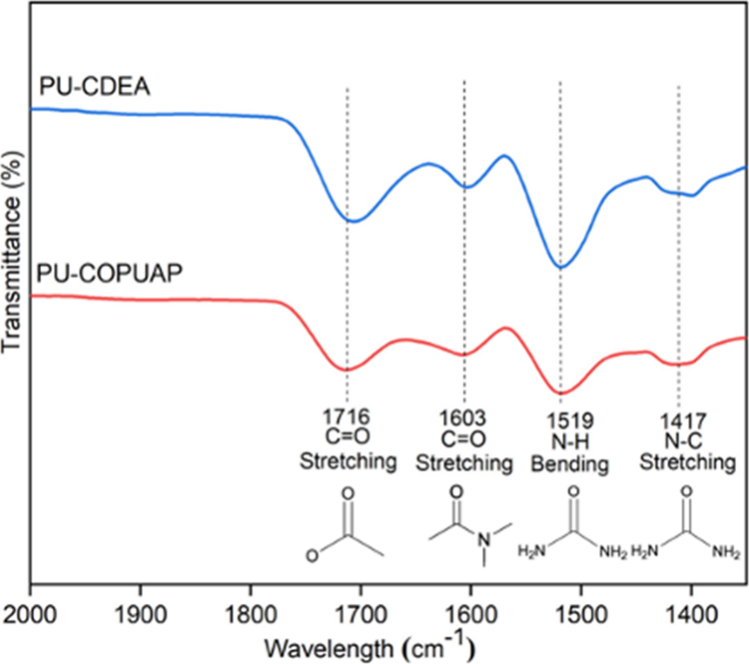
FTIR spectra of coconut oil polyurethane-urea prepolymer-based rigid foam (PU-COPUAP) and coconut diethanolamide-based rigid foam (PU-CDEA).

Another occurrence of cross-linking is evident in the FTIR spectra for both PU-CDEA and PU-COPUAP, which are shown in Figure 6. In PU-CDEA, the stretching vibration peak of urethane-based N–H bending vibration at around 1519 cm^–1^ and the C–N stretch vibration at 1417 cm^–1^ attributed to urea is already noticeable.^26^ Cross-linking which led to the consumption of the urea-based C–N group during foaming resulted in a decrease in the intensity of 1417 cm^–1^ of COPUAP.^26^ PU-CDEA exhibited an increase in the N–H bending vibration at 1519 cm^–1^, indicating the formation of urea.^26^ The decrease observed at 1603 cm^–1^, representing the C=O in urethane and amide groups, indicates its participation in cross-linking during foaming.^16,42^ Furthermore, a widening on the right side of the curve in the 1670–1630 cm^–1^ region of the FTIR spectrum of PU-CDEA indicates the presence of urea.^26^

**Figure 7:**
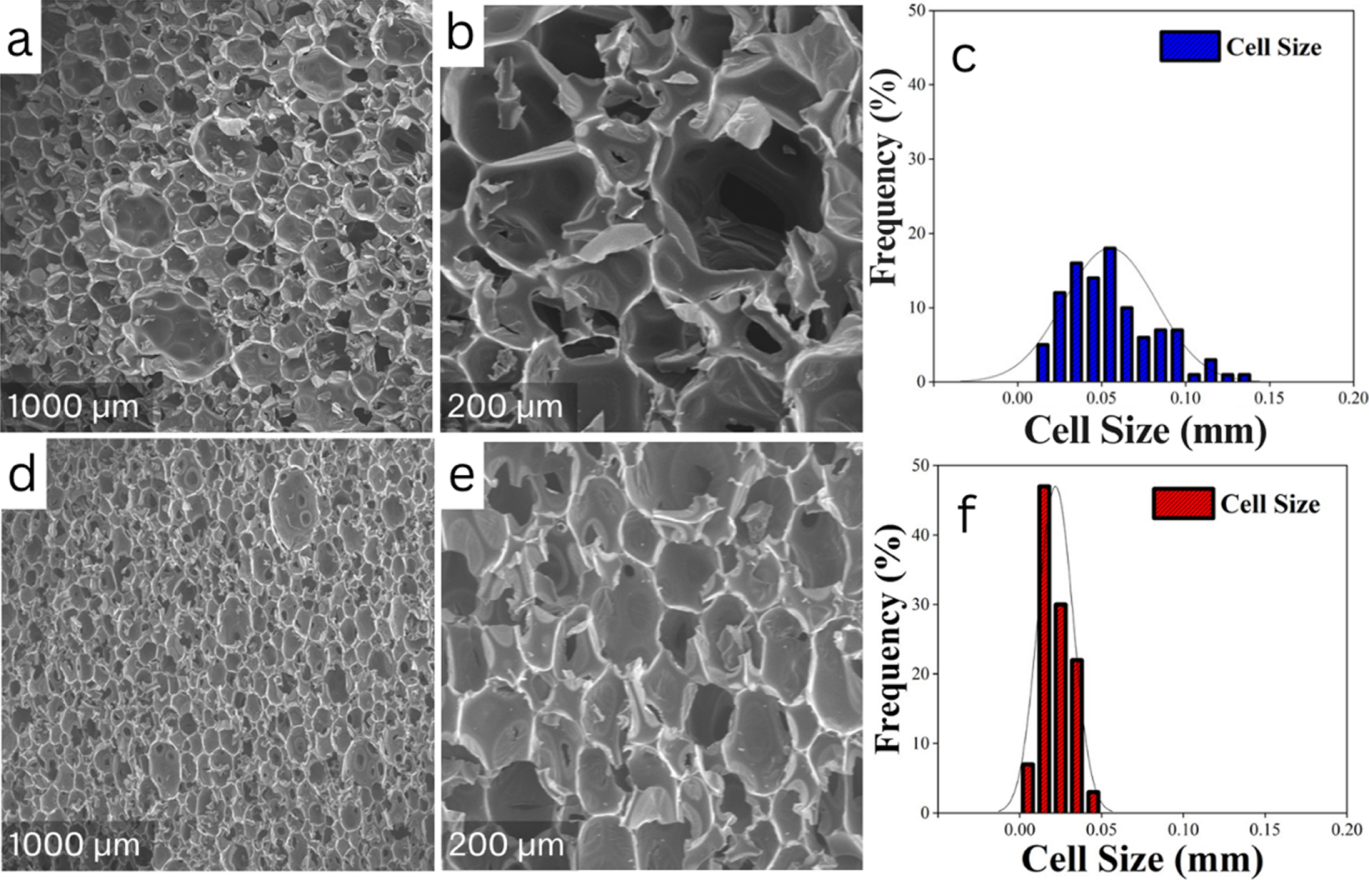
(a) SEM images of the external surface of (a) coconut diethanolamide-based rigid foam (PU-CDEA) 1000× magnification, (b) PU-CDEA 200x, (c) PU-CDEA cell size distribution, (d) coconut oil polyurethane-urea prepolymer-based rigid foam (PU-COPUAP) 1000×, (e) PU- COPUAP 200×, and (f) PU-COPUAP cell size distribution.

SEM images of PU-CDEA (Figure 7a,b) and PU-COPUAP (Figure 7d,e) revealed that PU-CDEA exhibited more ripped cells with irregular sizes and shapes compared to PU-COPUAP. The occurrence of cell rupture in PU-CDEA can be linked to its weak cell wall, which can compromise cell stability during bubble expansion.^48,49^ The exothermic nature of the reaction between *p*-CDEA and isocyanate during foaming (as shown in Figure 5a) results in an insufficient time for cell walls to stabilize, causing them to rupture. This reaction is responsible for the cell wall to fall apart as observed.

To address the adverse effects of the autocatalytic reaction of amino ester and isocyanate during the foaming process, incorporating a prepolymerization step limits the temperature increase (Figure 5a), as discussed earlier. Furthermore, it improves the cellular properties of the resulting foam by promoting chain lengthening capable of cross-linking during foaming.^50^ This prepolymerization process can serve to control the length propagation of urethane chains, leading to a reduction in cell wall rupturing and an improvement in both the mechanical and thermal properties of the foam.

The cell size distributions of both foams are shown in Figure 7c,f. PU-COPUAP exhibits a significantly smaller average cell size of 20.00 ± 1.20 μm compared to that of PU-CDEA with an average size of 48.00 ± 1.80 μm. This reduction in cell sizes is attributed to an increase in the degree of cross-linking during foaming, which promotes a suppression effect on foam cell coalescence.^34^ Notably, PU-COPUAP shows a relatively narrow and more uniform cell size distribution in comparison to that of PU-CDEA.

The presence of large cells in PU-COPUAP may be attributed to low-functional *p*-CDEA in COPUAP during foaming, as evident in the minimal traces identified in the FTIR result (Figure 6) of PU-COPUAP, albeit significantly less than in PU-CDEA. Moreover, extending the PU-urea prepolymer chain results in increased viscosity of the COPUAP polyol,^30,44^ which can adversely affect the uniform mixing of the A-side and B-side components during the foaming process. The narrow cell size distribution of COPUAP implies that the cells become more rigid and are less likely to merge and coalesce extensively. This can be attributed to a controlled foam-forming reaction rate and increased chemical bonding due to cross-linking, resulting in a more stable and consistent foam structure.^65^ The rigid cross-linking properties during the foaming process of PU-COPUAP restrain cell growth, which results in a higher foam density and closed cell composition. The average density of COPUAP increased remarkably from 57.1 ± 2.9 to 93.5 ± 1.2 kg/m^3^ relative to PU-CDEA. All of these findings are presented in Table 4.

**Table 4:** Physical, Mechanical, and Insulative Properties of Poly(urethane-urea) Rigid Foams

foam sample	density (kg/m^3^)	closed cell (%)	mean cell size (μm)	compressive strength (kPa)	flexural strength (kPa)	thermal conductivity (mW/(m·K))
PU-CDEA	57.1 ± 2.9	57.08 ± 0.1	48.00 ± 1.80	132 ± 15	232 ± 12.2	48.02 ± 3.5
PU-COPUAP	93.5 ± 1.2	79.97 ± 1.4	20.00 ± 1.20	628 ± 15	828 ± 13.5	34.52 ± 2.4
PU-V490	46.1 ± 1.5	92.98 ± 0.3	103.0 ± 2.0	723 ± 15	1157 ± 12.6	21.60 ± 0.3

### Physical Performance of PUA Rigid Foams (PU-CDEA and PU-COPUAP)

The evaluation of the mechanical properties of foam is crucial in providing information on the material’s ability to withstand compression and bending. To investigate the effect of prepolymerization on the mechanical performance of the foam, compressive and flexural tests were conducted. The compressive test measures the ability of the foam to withstand compressive loads through the application of an increasing compressive force until the foam breaks. The flexural test applies a bending force to the foam sample, measuring its resistance to bending or breaking. Table 4 summarizes the properties (density, % closed cell composition, cell size, and compressive and flexural strengths) of the rigid foam at 100% biobased polyol replacement.

**Figure 8:**
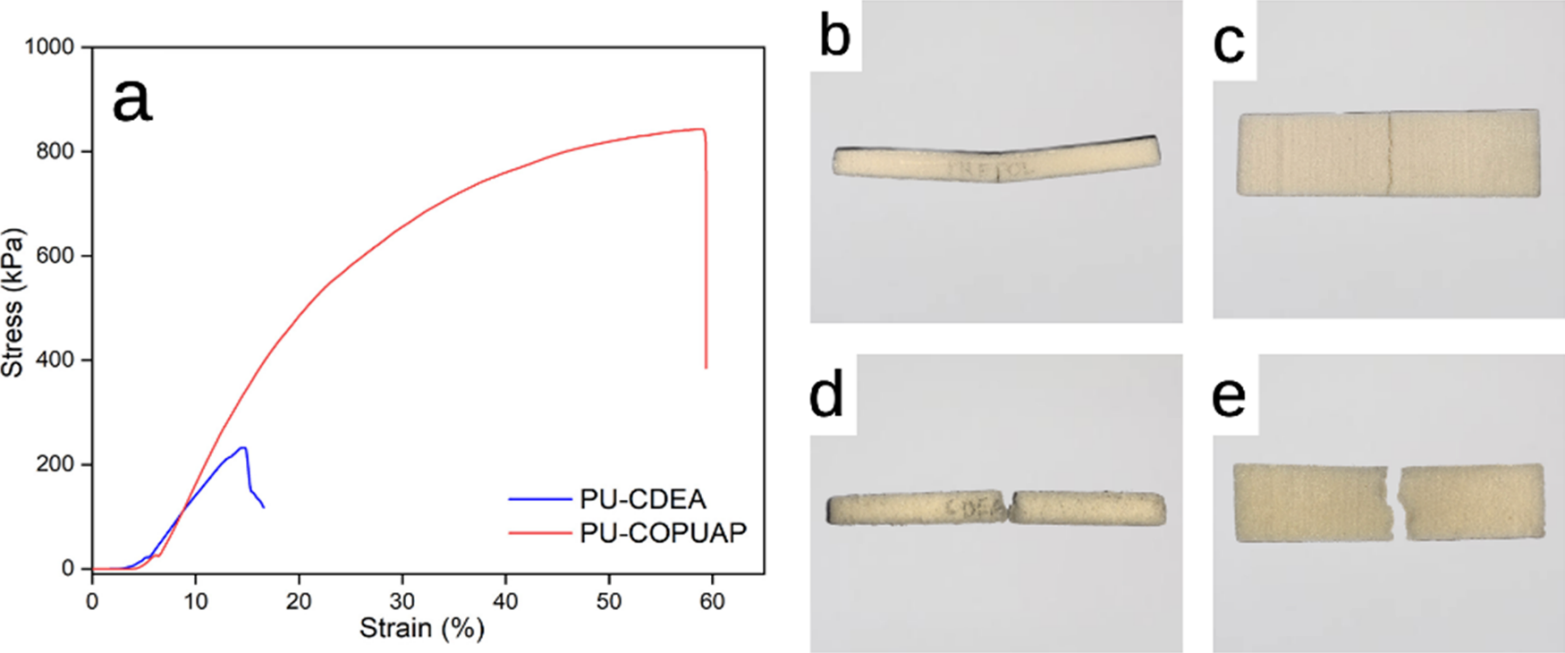
(a) Flexural test stress vs strain curves of coconut diethanolamide-based rigid polyurethane-urea foam (PU-CDEA) and coconut oil polyurethane-urea prepolymer rigid foam (PU-COPUAP); actual images after flexural testing: (b) side-view of PU-COPUAP, (c) top-view of PU-COPUAP, (d) side-view of PU-CDEA, and (e) top-view of PU-CDEA.

It is evident in Figure 8a that PU-COPUAP exhibited robustness by withstanding an ultimate stress of 820 kPa without completely breaking, whereas PU-CDEA experienced a complete break and separated into two pieces when it reached 230 kPa (Figure 8d). Figure 8b revealed that PU-COPUAP experienced a fracture but its upper surface remained structurally sound, highlighting its relatively superior strength and durability.

As indicated in Table 4, the compressive and flexural properties of PU-COPUAP rigid foam have shown 4.7- and 3.5-fold increases, respectively, relative to PU-CDEA. This result is consistent with the general trend observed in the cell morphology analysis (Figure 7a–e). It indicates that the morphological property has a great influence on the mechanical characteristics of rigid foam. Less ruptured cell walls, homogeneous structure, a greater number of cells per unit volume, and high-density characteristics of PU-COPUAP contribute to its strong mechanical properties.

The influence of the polymer matrix on the compression strength was further observed in the case of PU-V490 (Figure S3), which despite having a larger cell size compared to the biobased PUs, displayed the highest compressive strength. Nonetheless, a fully biobased PU-COPUAP exhibited a compressive strength closely comparable to that of petroleum-based rigid foam (Figure S5). All these findings have important implications for the development of biobased rigid foam materials with enhanced mechanical properties.

### Thermal Characterization of PU-CDEA and PU-COPUAP Foams

**Figure 9:**
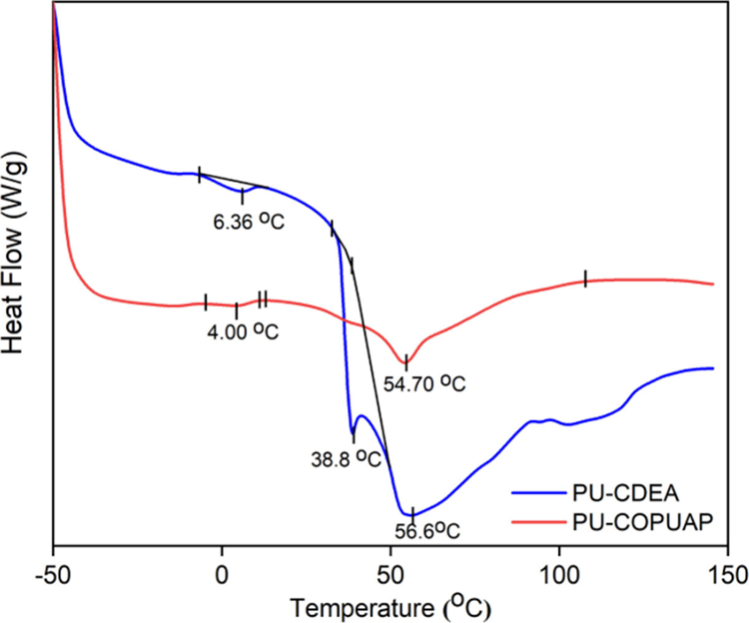
Differential scanning calorimetric thermogram of coconut diethanolamide-based polyurethane-urea rigid foam (PU-CDEA) and coconut oil polyurethane-urea prepolymer-based rigid foam (PU-COPUAP) showing thermal glass transition.

Understanding the thermal properties of foam is crucial in optimizing its processing conditions and applications.^51^ To investigate the thermal behavior, decomposition temperatures, and heat flow characteristics, the produced foams were analyzed using TGA and DSC techniques. As shown in the DSC thermograms in Figure 9, both PU-CDEA and PU-COPUAP samples show a closely similar glass transition temperature (*T*_g_) of 54.7 and 56.6 °C, respectively. These transition temperatures show the breaking of hydrogen bonds that takes place at the glass transition temperature (*T*_g_) of urethane segments.^23^ The *T*_g_ at 6.36 and 4.00 °C corresponds to the hydrogen dissociation of glycol content of both foams.^30^ Moreover, PU-CDEA reveals a unique peak at 38 °C, which is not present in the PU-COPUAP thermogram. According to Dingcong et al., this transition is attributed to *T*_g_ of urea segments.^14^ This observation was further confirmed by conducting a DSC analysis on the MDI and DEA reaction producing urea segments, which resulted in peaks at 38 and 101.7 °C that represent the dissociation of hydrogen bond of urea and MDI (Figure S4).^52^

Based on the PU-COPUAP DSC thermogram results, the foam displays a well-defined peak,^50^ which suggests that cross-linking in the COPUAP foam contributes to obtaining a uniformly formed macromolecule, in contrast to the PU-CDEA foam which showed multiple peaks. The consistency of cross-linking throughout the material is supported by the results presented in Figure 9 for PU-COPUAP.

**Figure 10:**
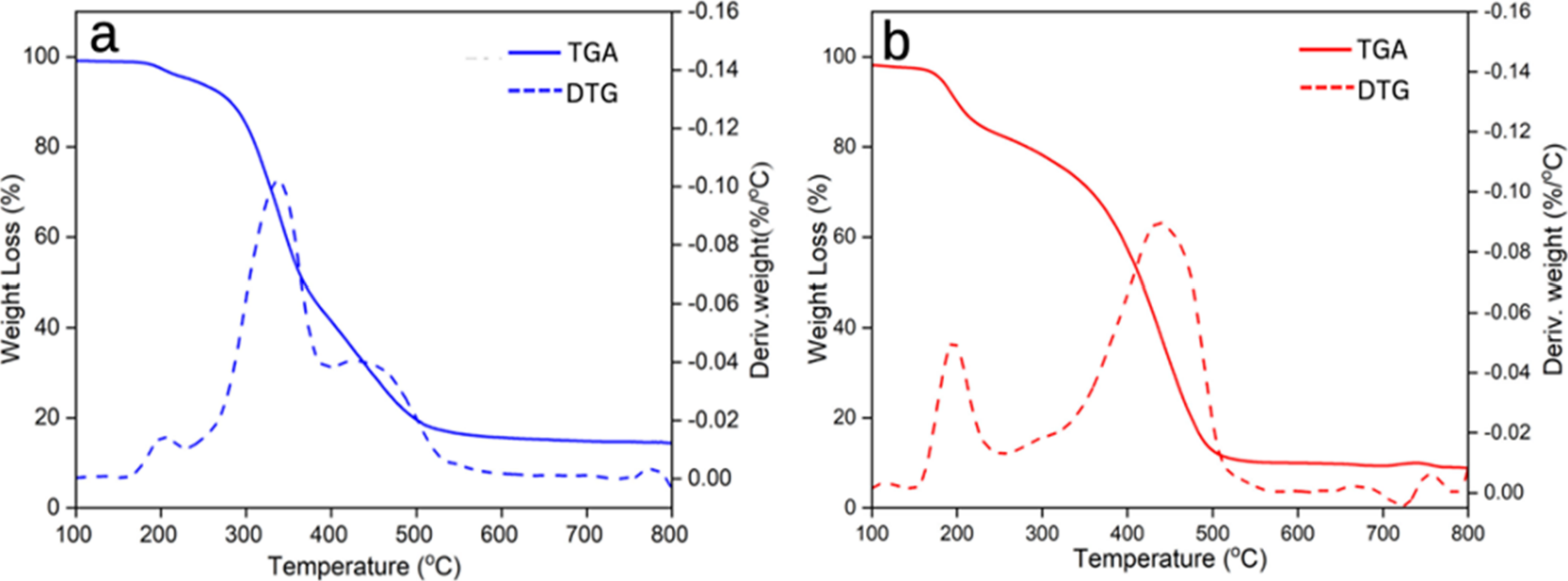
Weight loss (%) vs temperature (°C) for the (a) coconut diethanolamide-based rigid foam (PU-CDEA) and (b) coconut oil polyurethane-urea polyol-based rigid foam (PU-COPUAP) as represented by TGA and DTG curves.

Figure 10 shows the thermal gravimetric analysis (TGA) results of PU-COPUAP and PU-CDEA. PU-CDEA demonstrates several degradation peaks, as shown in Figure 10a. The first peak observed at 150–250 °C corresponds to the thermal degradation of hard segments such as urethane and urea linkages, resulting in the dissociation of alcohol and isocyanate as well as amino esters.^64^ Meanwhile, the second peak for PU-CDEA at 300–400 °C is attributed to the breaking of polymer chains, and the third peak at 510 °C corresponds to the thermal decomposition of soft segments in polyol and polyurea.^55−57^ For PU-COPUAP, the peak observed at 202 °C represents the decomposition of urethane bonds, while the peak at 300 °C signifies the decomposition of urea bonds.^55^ The peak around 354 and 508 °C corresponds to the degradation of a soft segment consisting of polyols and polyurea.^55,56^

The thermal behavior of the foam was significantly influenced by both the cell structure and the constituent component.^31^ As presented in Figure 10, the decomposition temperature (*T*_d_) at 5% weight loss (*T*_d_5) was found to be associated with the decomposition of hard segments,^29,56^ while the *T*_d_ at 50% weight loss (*T*_d_50) was predominantly linked to the degradation of soft segment structures. PU-CDEA has a lower *T*_d_50 of 375.38 °C compared to PU-COPUAP, which is 418 °C (Table S2). It signifies that PU-COPUAP is more thermally stable at higher temperatures compared to PU-CDEA. This is due to the combination of strong cross-linking,^56,63^ cell structure, and chain length of PU-COPUAP. Moreover, the thinner cell walls in PU-CDEA might have caused its cellular structures to collapse more easily at lower temperatures compared to PU-COPUAP, which coincides with the findings of Gu et al.^57^

The addition of prepolymerization led to a decrease in the thermal conductivity. Specifically, the thermal conductivity of PU-CDEA (*k* = 48.02 ± 3.5 mW/m·K) was found to be higher than that of PU-COPUAP (*k* = 34.52 ± 2.4 mW/m·K). This can be attributed to the presence of voids resulting from ruptured cell walls which can act as a pathway for heat transfer, allowing for air movement and heat transfer through convection.^58^^,^^59^ Consequently, this can increase the measured heat flow and result in additional heat transfer from the material. Foams with rigid cells have a lower likelihood of cell rupture and do not allow air to flow through, thus aiding in minimizing heat transfer through convection and improving insulation capability.^60^ Thicker cell walls can also create greater resistance to the flow of heat, reducing thermal conductivity and enhancing the insulation properties of the foam. Thus, achieving a minimized number of ruptured cells and enhanced morphological properties through prepolymerization has significant implications in reducing heat conductivity, which translates to its potential to function as an effective heat insulation system.

### Hydrophobic Properties of the Prepared Rigid Foams

To improve the overall foam’s shelf life, the foam must possess a certain hydrophobic characteristic for enhanced shape fidelity and durability against environmental factors such as water/moisture. Recent studies show that typical water contact angles for biobased rigid foams range from 115°–140°.^61−63^

**Figure 11:**
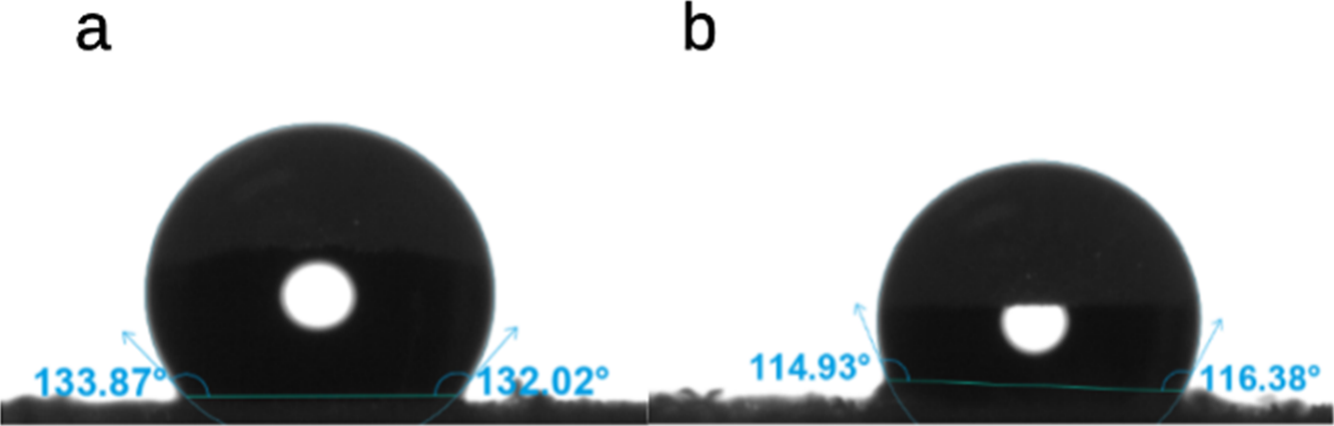
Water contact angles of (a) coconut oil polyurethane-urea prepolymer polyol-based rigid foam (PU-COPUAP) and (b) coconut diethanolamide-based rigid foam (PU-CDEA).

A water contact angle study has shown that both the PU-CDEA and PU-COPUAP foams have hydrophobic characteristics. As demonstrated in Figure 11, the addition of the prepolymerization process increased the water contact angle from 114.93° (PU-CDEA) to 133.87° (PU-COPUAP), representing an average contact angle increase of 16.5%. The hydrophobic property of CO-based RPUAF is attributed to its chemical and structural composition.^12^ In addition to its hydrophobic nature, which is attributed to the intrinsic C–H bond components,^66^ the presence of hydrophilic urea segments affects the foam’s hydrophobicity as a whole. Urea contains two polar amide groups which can form hydrogen bonds with water.^67^ The oxygen and nitrogen atoms in the amide groups have partial negative and positive charges, respectively, which allows them to form hydrogen bonds with the partially positive and negative charges on water molecules.^67^ As indicated earlier, PU-CDEA contains more hydrophilic urea compared with PU-COPUAP, as it was already utilized for biuret formation. Prepolymerization ensures the formation of urea, allowing for a direct progression to biuret formation during cross-linking in foam. As a result, the presence of urea in PU-COPUAP is minimized thus improving the foam’s hydrophobicity.^68^

Moreover, the cross-linked structure creates a network of tightly packed molecules (as shown in SEM images in Figure 7 b-e) with fewer gaps for water to penetrate, reducing the foam’s ability to absorb water. Furthermore, the cross-linked structure of PU-COPUAP creates a more complex and convoluted surface, making it difficult for water molecules to adhere to the foam. Finally, the tighter structure of the PU-COPUAP reduces the amount of free space available for water to accumulate, further limiting its ability to penetrate and be absorbed by the foam.^69,73^ All of these factors combined make PU-COPUAP relatively more hydrophobic.

Hydrophobic RPUAF can be used in a variety of metallic applications including the insulation of pipelines and tanks. Hydrophobic rigid foams can be used to insulate pipelines and tanks used in the transportation and storage of corrosive and high-temperature fluids. It prevents the metal surface from being exposed to moisture, and at the same time, it provides insulation and protection against corrosion in such environments.^70,71^ It can also be used in marine and offshore structures and protect metal surfaces on offshore structures such as water board fill and floatation devices. The foam’s ability to resist water absorption and its durability make it an ideal material for such applications.^72^ Overall, these results provide compelling evidence of the excellent water and moisture resistance exhibited by RPUAFs based on a COPUAP.

## Conclusions

In this investigation, COPUAP was successfully synthesized utilizing a sequential amidation-prepolymerization methodology, leading to the production of polyols, namely, *p*-CDEA and COPUAP. Characterization of these polyols involved a series of characterization including viscosity, OHV, NHV, and FTIR analyses. The confirmation of chain lengthening in COPUAP polyol was evident through a substantial increase in viscosity (from 472.4 to 755.8 mPa·s). The observed 98% rise in NHV was attributed to the continuous formation of amine groups, contributing to the cross-linking of COPUAP, while a decrease in OH functionality (from 496.3 to 473.2 mg KOH/g) was linked to the consumption of OH functionalities, indicative of cross-linking in previously reactive functional groups. As sole B-side polyol components in foam formulations, COPUAP and *p*-CDEA were compared for their morphological, thermal, and mechanical properties. SEM images highlighted that the COPUAP-based foam exhibited reduced cell rupture and deformation during expansion, signifying thicker cell walls. The PU-COPUAP foam demonstrated improved compressive and flexural properties, with a 4.7-fold (132–628 kPa) and a 3.5-fold (232–828 kPa) increase, respectively, relative to PU-CDEA. Furthermore, the enhanced thermal insulation (34.52 ± 2.4 mW/m·K) and heightened hydrophobicity (133° contact angle) of PU-COPUAP position it as a promising material for building insulation, load-bearing applications, and industrial usage in corrosive metallic environments, expanding its potential in marine and offshore structures as well as for protecting pipelines and tanks. The simplicity of COPUAP polyol’s production process further underscores its suitability for practical applications.

## Supporting Information Available

Mechanical and thermal properties of foams, digital images of experimentation, DSC thermogram, and SEM images (PDF)

## Abbreviations

RPUAF: rigid polyurethane-urea foam
*p*-CDEA: coconut diethanolamide
COPUAP: coconut oil poly(urethane-urea) prepolymer

## References

[ref1] Kumbar S; Laurencin C. T.; Deng M. Natural and synthetic biomedical polymers. essay; Elsevier, 2014.

[ref2] Król P.; Pilch-Pitera B. Mechanical properties of crosslinked polyurethane elastomers based on well-defined prepolymers. J. Appl. Polym. Sci. [Internet]. 2008, 107 (3), 1439–1448. 10.1002/app.26482.

[ref3] Shelke N; Nagarale R; Kumbar D. Polyurethanes. In Natural and synthetic biomedical polymers; Elsevier: Amsterdam, 2014.

[ref4] Heath D. E.; Guelcher S. A.; Cooper S. L. Polyurethanes. In Biomaterials Science; Wagner W. R.; Sakiyama-Elbert S. E.; Zhang G; Yaszemski M. J., Eds.; Elsevier: San Diego, CA; 2020; pp 103–107.

[ref5] Grünbauer H.; Bicerano C.; Daussin R.; de Vos H.; Elwell M. Rigid Polyurethane Foams. In Polymeric Foams; CRC Press, 2004.

[ref6] Paruzel A.; Michałowski S.; Hodan J.; Horák P.; Prociak A.; Beneš H. Rigid polyurethane foam fabrication using medium chain glycerides of coconut oil and plastics from end-of-life vehicles. ACS Sustain Chem. Eng. [Internet]. 2017, 5 (7), 6237–6246. 10.1021/acssuschemeng.7b01197.

[ref7] Mohanty A. K.; Misra M.; Drzal L. T. Biobased polyurethanes and their composites: Present status and future perspective. In Natural Fibers, Biopolymers, and Biocomposites; CRC Press, 2005.

[ref8] Lligadas G.; Ronda J. C.; Galià M.; Cádiz V. Renewable polymeric materials from vegetable oils: a perspective. Mater. Today (Kidlington) [Internet]. 2013, 16 (9), 337–343. 10.1016/j.mattod.2013.08.016.

[ref9] Petrović Z. S.; Zhang W.; Javni I. Structure and properties of polyurethanes prepared from triglyceride polyols by ozonolysis. Biomacromolecules [Internet]. 2005, 6 (2), 713–719. 10.1021/bm049451s. 15762634

[ref10] Yeganeh H.; Hojati-Talemi P. Preparation and properties of novel biodegradable polyurethane networks based on castor oil and poly(ethylene glycol). Polym. Degrad. Stab. 2007, 92 (3), 480–489. 10.1016/j.polymdegradstab.2006.10.011.

[ref11] Zieleniewska M.; Leszczyński M. K.; Kurańska M.; Prociak A.; Szczepkowski L.; Krzyżowska M.; et al. Preparation and characterization of rigid polyurethane foams using a rapeseed oil-based polyol. Ind. Crops Prod [Internet]. 2015, 74, 887–897. 10.1016/j.indcrop.2015.05.081.

[ref12] Stirna U.; Lazdiṇa B.; Vilsone D.; Lopez M. J.; Vargas Garcia M.; Del C.; Suárez Estrella F.; et al. Structure and properties of the polyurethane and polyurethane foam synthesized from castor oil polyols. J. Cell. Plast. 2012, 48 (6), 476–488. 10.1177/0021955x12445178.

[ref13] Carme Coll Ferrer M.; Babb D.; Ryan A. J. Characterisation of polyurethane networks based on vegetable derived polyol. Polymer (Guildf) [Internet]. 2008, 49 (15), 3279–3287. 10.1016/j.polymer.2008.05.017.

[ref14] Lee J. H.; Kim S. H.; Oh K. W. Bio-Based Polyurethane Foams with Castor Oil Based Multifunctional Polyols for Improved Compressive Properties. Polymers 2021, 13, 576 10.3390/polym13040576. 33672983 PMC7918616

[ref15] Hejna A.; Kirpluks M.; Kosmela P.; Cabulis U.; Haponiuk J.; Piszczyk Ł. The Influence of Crude Glycerol and Castor Oil-Based Polyol on the Structure and Performance of Rigid Polyurethane-Polyisocyanurate Foams. Industrial Crops and Products 2017, 95, 113–125. 10.1016/j.indcrop.2016.10.023.

[ref16] Leng X.; Li C.; Cai X.; Yang Z.; Zhang F.; Liu Y.; et al. A study on coconut fatty acid diethanolamide-based polyurethane foams. RSC Adv. [Internet]. 2022, 12 (21), 13548–13556. 10.1039/D2RA01361D. 35527733 PMC9069328

[ref17] Coconut trade performance and market. https://pca.gov.ph/images/pdf/2022_Coconut_Trade_Performance_and_Market_Trends.pdf (accessed January 2024).

[ref18] Salcedo Ma L.; Omisol C. J.; Maputi A. O.; Estrada D. J.; Aguinid B. J.; Asequia D. M.; Erjeno D. J.; Apostol G.; Siy H.; Malaluan R. M. Production of bio-based polyol from coconut fatty acid distillate (CFAD) and crude glycerol for rigid polyurethane foam applications; 2023. 10.3390/ma16155453 PMC10420174 37570156

[ref19] Omisol C. J.; Aguinid B. J.; Abilay G. Y.; Asequia D. M.; Tomon T. R.; Sabulbero K. X.; Erjeno D. J.; Osorio C. K.; Usop S.; Malaluan R. Flexible Polyurethane Foams Modified with Novel Coconut Monoglycerides-Based Polyester Polyols. ACS Omega 2024, 9, 4497–4512. 10.1021/acsomega.3c07312. 38313545 PMC10831968

[ref20] Asare M. A.; de Souza F. M.; Gupta R. K. Waste to Resource: Synthesis of Polyurethanes from Waste Cooking Oil. ACS Industrial Engineering Chemistry Research 2022, 61, 18400–18411. 10.1021/acs.iecr.2c03718.

[ref21] Khanmohammadi M.; Kojidi M. H.; Garmarudi A. B.; Ashuri A.; Soleymani M. Quantitative monitoring of the amidation reaction between coconut oil and diethanolamine by attenuated total reflectance Fourier transform infrared spectrometry. J. Surfactants Deterg [Internet]. 2009, 12 (1), 37–41. 10.1007/s11743-008-1101-7.

[ref22] Veronese V. B.; Menger R. K.; Forte M. M. d. C.; Petzhold C. L. Rigid polyurethane foam based on modified vegetable oil. J. Appl. Polym. Sci. 2011, 120 (1), 530–537. 10.1002/app.33185.

[ref23] Dingcong R. G. Jr; Malaluan R. M.; Alguno A. C.; Estrada D. J. E.; Lubguban A. A.; Resurreccion E. P.; et al. A novel reaction mechanism for the synthesis of coconut oil-derived Biopolyol for rigid poly(urethane-urea) hybrid foam application. RSC Adv. [Internet]. 2023, 13 (3), 1985–1994. 10.1039/D2RA06776E. 36712635 PMC9832577

[ref24] Liang H.; Li Y.; Huang S.; Huang K.; Zeng X.; Dong Q.; et al. Tailoring the performance of vegetable oil-based waterborne polyurethanes through incorporation of rigid cyclic rings into soft polymer networks. ACS Sustain Chem. Eng. [Internet]. 2020, 8 (2), 914–925. 10.1021/acssuschemeng.9b05477.

[ref25] Swamy B. K.; Hatna S.; Rudrappa S. Structure-property relationship of castor oil-based diol chain extended polyurethanes: Semantic scholar. J. Mater. Sci. 1970, 38, 451–460. 10.1023/A:1021815514141.

[ref26] Gurunathan T.; Mohanty S.; Nayak S. K. isocyanate terminated castor oil-based polyurethane prepolymer: Synthesis and characterization.. Prog. Org. Coat. [Internet] 2015, 80, 39–48. 10.1016/j.porgcoat.2014.11.017.

[ref27] Badri K. B. H.; Sien W. C.; Shahrom MSBR; Hao L. C.; Baderuliksan N. Y.; Norzali N. R. A. Biobased polyurethane from palm kernel oil-based Polyol. Solid State Sci. Technol. 2012, 18, 1–8.

[ref28] Prabhakar A.; Chattopadhyay D. K.; Jagadeesh B.; Raju K. V. S. N. Structural investigations of polypropylene glycol (PPG) and isophorone diisocyanate (IPDI)-based polyurethane prepolymer by 1D and 2D NMR spectroscopy. J. Polym. Sci., A: Polym. Chem. 2005, 43 (6), 1196–1209. 10.1002/pola.20583.

[ref29] Xie D.-M.; Zhang Y.-X.; Li Y.-D.; Weng Y.; Zeng J.-B. Castor oil-derived sustainable poly(urethane urea) covalent adaptable networks with tunable mechanical properties and multiple recyclability based on reversible piperidine-urea bond. Chem. Eng. J. 2022, 446, 137071 10.1016/j.cej.2022.137071.

[ref30] Mora-Murillo L. D.; Orozco-Gutierrez F.; Vega-Baudrit J.; González-Paz R. J. Thermal-mechanical characterization of polyurethane rigid foams: Effect of modifying bio-polyol content in isocyanate prepolymers. J. Renew Mater. [Internet]. 2017, 5 (3), 220–230. 10.7569/JRM.2017.634112.

[ref31] Sriram V.; Mahesh G. N.; Jeevan R. G.; Radhakrishnan G. Comparative studies on short-chain and long-chain crosslinking in polyurethane networks.. Macromol. Chem. Phys. 2000, 201 (18), 2799–2804. 10.1002/1521-3935(20001201)201:18<2799::aid-macp2799>3.0.co;2-g.

[ref32] Saad N. M.; Salleh N. M.; Abdullah T. K.; Zubir S. A. Influence of prepolymer reaction time in the fabrication of palm kernel oil polyol based shape memory polyurethane. J. Appl. Polym. Sci. 2022, 139 (19), 52109 10.1002/app.52109.

[ref33] Bernardini J.; Licursi D.; Anguillesi I.; Cinelli P.; Coltelli M.-B.; Antonetti C.; et al. Exploitation of Arundo donax L. hydrolysis residue for the Green synthesis of flexible polyurethane foams. Bioresources [Internet]. 2017, 12, 2 10.15376/biores.12.2.3630-3655.

[ref34] Zhai W.; Wang H.; Yu J.; Dong J.; He J. Cell coalescence suppressed by crosslinking structure in polypropylene microcellular foaming. Polym. Eng. Sci. [Internet]. 2008, 48 (7), 1312–1321. 10.1002/pen.21095.

[ref35] Cha S. W.; Cho S.; Sohn J. S.; Ryu Y.; Ahn J. Reflectance According to Cell Size, Foaming Ratio and Refractive Index of microcellular Foamed Amorphous Polymer. International Journal of Molecular Sciences 2019, 20, 6068 10.3390/ijms20236068. 31810176 PMC6928872

[ref36] Pongmuksuwan P.; Harnnarongchai W. Synthesis and Characterization of Soft Polyurethane for Pressure Ulcer Prevention. Polym. Test. 2022, 112, 107634 10.1016/j.polymertesting.2022.107634.

[ref37] Gogoi P.; Boruah M.; Bora C.; Dolui S. K. Jatropha Curcas Oil Based Alkyd/Epoxy Resin/Expanded Graphite (EG) Reinforced Bio-Composite: Evaluation of the Thermal, Mechanical and Flame Retardancy Properties. Prog. Org. Coat. 2014, 77, 87–93. 10.1016/j.porgcoat.2013.08.006.

[ref38] Sun S.; Dai C.; Sun L.; Seh Z. W.; Sun Y.; Fisher A.; et al. The effect of the hydroxyl group position on the electrochemical reactivity and product selectivity of butanediol electro-oxidation. Dalton Trans [Internet]. 2022, 51 (38), 14491–14497. 10.1039/D2DT02450K. 36106440

[ref39] Herington R. Dow’s book on Flexible Polyurethane Foams; The Dow Chemical Company: United States, 1997.

[ref40] Baghban S.; Khorasani M.; Sadeghi M. M. Soundproofing flexible polyurethane foams: Effect of chemical structure of chain extenders on micro-phase separation and acoustic damping. J. Cell. Plast. 2020, 56 (2), 167–185. 10.1177/0021955x19864387.

[ref41] Bocchio J. A.; Escobar M. M.; Quagliano Amado J. C. Ablative Properties of Polyurethanes Reinforced with Organoclay. Polymer Engineering &amp; Science 2020, 60, 630–635. 10.1002/pen.25321.

[ref42] Cheikh W.; Rózsa Z. B.; Camacho López C. O.; Mizsey P.; Viskolcz B.; Szőri M.; et al. Urethane formation with an excess of isocyanate or alcohol: Experimental and ab initio study. Polymers (Basel) [Internet]. 2019, 11 (10), 1543 10.3390/polym11101543. 31546721 PMC6835639

[ref43] Giroto A. S.; Valle S. F.; Ribeiro T.; Ribeiro C. Towards urea and glycerol utilization as “building blocks” for polyurethane production: A detailed study about reactivity and structure for environmentally friendly polymer synthesis. React. Funct. Polym. 2020, 153, 104629 10.1016/j.reactfunctpolym.2020.104629.

[ref44] Fukushima K.; Yaguchi H. Development of high performance water-blown polyurethane rigid foams. Polym. J. 1998, 30 (8), 633–642. 10.1295/polymj.30.633.

[ref45] Mahmoud A. A.; Nasr E. A. A.; Maamoun A. A. H. The influence of polyurethane foam on the insulation characteristics of mortar pastes. J. Miner Mater. Charact Eng. [Internet]. 2017, 05 (02), 49–61. 10.4236/jmmce.2017.52005.

[ref46] Dai C.; Zhao G.; You Q.; Zhao M.; Liu Y.; Zhao F. Preparation technology of Bulk Gel. Theory and Technology of Multiscale Dispersed Particle Gel for In-Depth Profile Control 2022, 11–45. 10.1016/B978-0-323-99849-9.00002-3.

[ref47] Shah A. J.; Parikh S. J.; Parikh M. H.; Patel S. K. Effect of reaction kinetics on the mechanical properties of polyurethane foams. J. Appl. Polym. Sci. 2020, 137, (12), .

[ref48] Zeng X.; Tang T.; An J.; Liu X.; Xiang H.; Li Y.; et al. Integrated preparation and properties of polyurethane-based sandwich structure composites with foamed core layer. Polym. Compos [Internet]. 2021, 42 (9), 4549–4559. 10.1002/pc.26167.

[ref49] Aliotta L.; Vannozzi A.; Canesi I.; Cinelli P.; Coltelli M.-B.; Lazzeri A. Poly(lactic acid) (pla)/poly(butylene succinate-co-adipate) (PBSA) compatibilized binary biobased blends: Melt fluidity, morphological, thermo-mechanical and Micromechanical Analysis. Polymers. 2021, 13 (2), 218 10.3390/polym13020218. 33435479 PMC7827856

[ref50] Borrero-López A. M.; Nicolas V.; Marie Z.; Celzard A.; Fierro V. A review of rigid polymeric cellular foams and their greener tannin-based alternatives. Polymers 2022, 14 (19), 3974 10.3390/polym14193974. 36235923 PMC9572835

[ref51] Zhu Y.; Zhu J.; Yu Z.; Ye Y.; Sun X.; Zhang Y. Air drying scalable production of hydrophobic, mechanically stable, and thermally insulating lignocellulosic foam. Chem. Eng. J. 2022, 450, 138300 10.1016/j.cej.2022.138300.

[ref52] Zhuang J. M.; Steiner P. R. Thermal reactions of diisocyanate (MDI) with phenols and benzylalcohols: DSC study and synthesis of MDI adducts. hfsg. 1993, 47 (5), 425–434. 10.1515/hfsg.1993.47.5.425.

[ref53] Tan M. W.; Thangavel G.; Lee P. S. Enhancing Dynamic Actuation Performance of Dielectric Elastomer Actuators by Tuning Viscoelastic Effects with Polar Crosslinking. NPG Asia Mater. 2019, 11, 62 10.1038/s41427-019-0147-5.

[ref54] Zhang Y.; Li Y.; Liu W. Dipole–Dipole and H-bonding Interactions Significantly Enhance the Multifaceted Mechanical Properties of Thermoresponsive Shape Memory Hydrogels. Adv. Funct. Mater. 2015, 25, 471–480. 10.1002/adfm.201401989.

[ref55] Shi A.; Zhang G.; Zhao C. Study of rigid cross-linked PVC foams with heat resistance. Molecules [Internet]. 2012, 17 (12), 14858–14869. 10.3390/molecules171214858. 23519258 PMC6268979

[ref56] Cao Z.-J.; Liao W.; Wang S.-X.; Zhao H.-B.; Wang Y.-Z. Polyurethane foams with functionalized graphene towards high fire-resistance, low smoke release, superior thermal insulation. Chem. Eng. J. [Internet]. 2019, 361, 1245–1254. 10.1016/j.cej.2018.12.176.

[ref57] Gu R.; Konar S.; Sain M. Preparation and characterization of sustainable polyurethane foams from soybean oils. J. Am. Oil Chem. Soc. [Internet]. 2012, 89 (11), 2103–2111. 10.1007/s11746-012-2109-8.

[ref58] Członka S.; Strąkowska A.; Kairytė A. Effect of walnut shells and silanized walnut shells on the mechanical and thermal properties of rigid polyurethane foams. Polym. Test. 2020, 87, 106534 10.1016/j.polymertesting.2020.106534.

[ref59] Ghosh I. Heat transfer correlation for high-porosity open-cell foam. Int. J. Heat Mass Transf [Internet]. 2009, 52 (5–6), 1488–1494. 10.1016/j.ijheatmasstransfer.2008.07.047.

[ref60] Ameli A.; Nofar M.; Jahani D.; Rizvi G.; Park C. B. Development of high void fraction polylactide composite foams using injection molding: Crystallization and foaming behaviors. Chem. Eng. J. [Internet]. 2015, 262, 78–87. 10.1016/j.cej.2014.09.087.

[ref61] Amri M. R.; Al-Edrus S. S. O.; Guan C. T.; Yasin F. M.; Hua L. S. Jatropha oil as a substituent for palm oil in biobased polyurethane. Int. J. Polym. Sci. 2021, 2021, 6655936 10.1155/2021/6655936.

[ref62] Sienkiewicz N.; Członka S.; Kairyte A.; Vaitkus S. Curcumin as a natural compound in the synthesis of rigid polyurethane foams with enhanced mechanical, antibacterial and anti-ageing properties. Polym. Test. 2019, 79, 106046 10.1016/j.polymertesting.2019.106046.

[ref63] Członka S.; Bertino M. F.; Kośny J.; Strąkowska A.; Masłowski M.; Strzelec K. Linseed oil as a natural modifier of rigid polyurethane foams. Ind. Crops Prod [Internet]. 2018, 115, 40–51. 10.1016/j.indcrop.2018.02.019.

[ref64] Septevani A. A.; Evans D. A. C.; Martin D. J.; Annamalai P. K. Hybrid polyether-palm oil polyester polyol based rigid polyurethane foam reinforced with cellulose nanocrystal. Ind. Crops Prod [Internet]. 2018, 112, 378–388. 10.1016/j.indcrop.2017.12.032.

[ref65] Chen N. The effects of crosslinking on foaming of eva [Internet]. TSpace. 2012. https://TSpace.library.utoronto.ca/handle/1807/32682.

[ref66] Oribayo O.; Feng X.; Rempel G. L.; Pan Q. Synthesis of lignin-based polyurethane/graphene oxide foam and its application as an absorbent for oil spill clean-ups and recovery. Chemical Engineering Journal. 2017, 323, 191–202. 10.1016/j.cej.2017.04.054.

[ref67] Politi M. J.; Chaimovich H.; Liu C.; Triboni E. R.; Briotto Filho D.; Cuccovia I. M. Effect of urea on ion pair formation. the hydrophilic effect of Urea. Colloids and Surfaces A: Physicochemical and Engineering Aspects. 2017, 520, 173–177. 10.1016/j.colsurfa.2017.01.068.

[ref68] Pikhurov D. V.; Sakhatskii A. S.; Zuev V. V. Rigid polyurethane foams with infused hydrophilic/hydrophobic nanoparticles: Relationship between cellular structure and physical properties. Eur. Polym. J. 2018, 99, 403–414. 10.1016/j.eurpolymj.2017.12.036.

[ref69] Sundar S.; Aruna P.; Venkateshwarlu U.; Radhakrishnan G. Aqueous dispersions of polyurethane cationomers: a new approach for hydrophobic modification and crosslinking. Colloid Polym. Sci. [Internet]. 2004, 283 (2), 209–218. 10.1007/s00396-003-1012-0.

[ref70] Dufour V., Ed.; Insulation solutions - huntsman-pimcore.equisolve-dev.com [Internet]. huntsman-pimcore; Huntsman Corporation, 2012. https://huntsman-pimcore.equisolve-dev.com/Documents/PU_Insulation_Insulation_solutions_2012.pdf.

[ref71] Uram K.; Prociak A.; Vevere L.; Pomilovskis R.; Cabulis U.; Kirpluks M. Natural oil-based rigid polyurethane foam thermal insulation applicable at cryogenic temperatures. Polymers. 2021, 13 (24), 4276 10.3390/polym13244276. 34960827 PMC8707178

[ref72] Crupi V.; Epasto G.; Napolitano F.; Palomba G.; Papa I.; Russo P. Green Composites for maritime engineering: A Review. Journal of Marine Science and Engineering. 2023, 11 (3), 599 10.3390/jmse11030599.

[ref73] Sambasivam M.; White R.; Cutting K. Exploring the role of polyurethane and polyvinyl alcohol foams in wound care. In Wound Healing Biomaterials; Elsevier, 2016; pp 251–260.

