# Supplementary material for: Development of High-Performance Coconut Oil-Based Rigid Polyurethane-Urea Foam: A Novel Sequential Amidation and Prepolymerization Process
Source: ACS Omega. 2024 Mar 7;9(11):13112–24. doi: 10.1021/acsomega.3c09598 (PMC10956093; doi:10.1021/acsomega.3c09598)
Supplement: Supplementary file 1 — ao3c09598_si_001.pdf [file ao3c09598_si_001.pdf]

Supplementary data

Development of high-performance coconut oil-based rigid polyurethane-urea foam: A novel sequential amidation and prepolymerization process

Louell Nikki A. Hipulan,[1,2,3] Roger G. Dingcong Jr.,[1] Dave Joseph E. Estrada,[1] Gerard G. Dumancas,[5] John Christian Bondaug,[1,2] Arnold C. Alguno,[1,6] Hernando P. Bacosa,[1,2] Roberto M. Malaluan,[1,4] and Arnold A. Lubguban*[1,4]

[1]Center for Sustainable Polymers, Mindanao State University – Iligan Institute of Technology, A. Bonifacio Avenue, 9200 Iligan City, Philippines; e-mail: arnold.lubguban@g.msuiit.edu.ph

[2]Environmental Science Graduate Program, Department of Biological Sciences, Mindanao State University – Iligan Institute of Technology, A. Bonifacio Avenue, 9200 Iligan City, Philippines

[3]College of Technology, University of San Agustin, General Luna St., 5000 Iloilo City, Philippines

[4]Department of Chemical Engineering and Technology, Mindanao State University – Iligan Institute of Technology A. Bonifacio Avenue, 9200 Iligan City, Philippines

[5]Department of Chemistry, The University of Scranton, Scranton, PA 18510 USA

[6] Department of Physics, Mindanao State University – Iligan Institute of Technology, A. Bonifacio Avenue, 9200 Iligan City, Philippines

Table S1. Mechanical and thermal properties of PU-CDEA and PU-COPUAP.

Sample	*E* (MPa)	σ (MPa)	ε (%)	$T_{5\%}$ (°C)	$T_{50\%}$ (°C)	T_g (°C)
PU-CDEA	0.264	0.232	0.88	243.50	375.38	6.36
PU-COPUAP	1.47	0.828	0.56	204.74	418.56	4.00
PU-V490	1.86	1.157	1.60	250.34	334.40	-4.30

T_g obtained from the DSC results.

$T_{5\%}$ *and* $T_{50\%}$ obtained from TGA results.

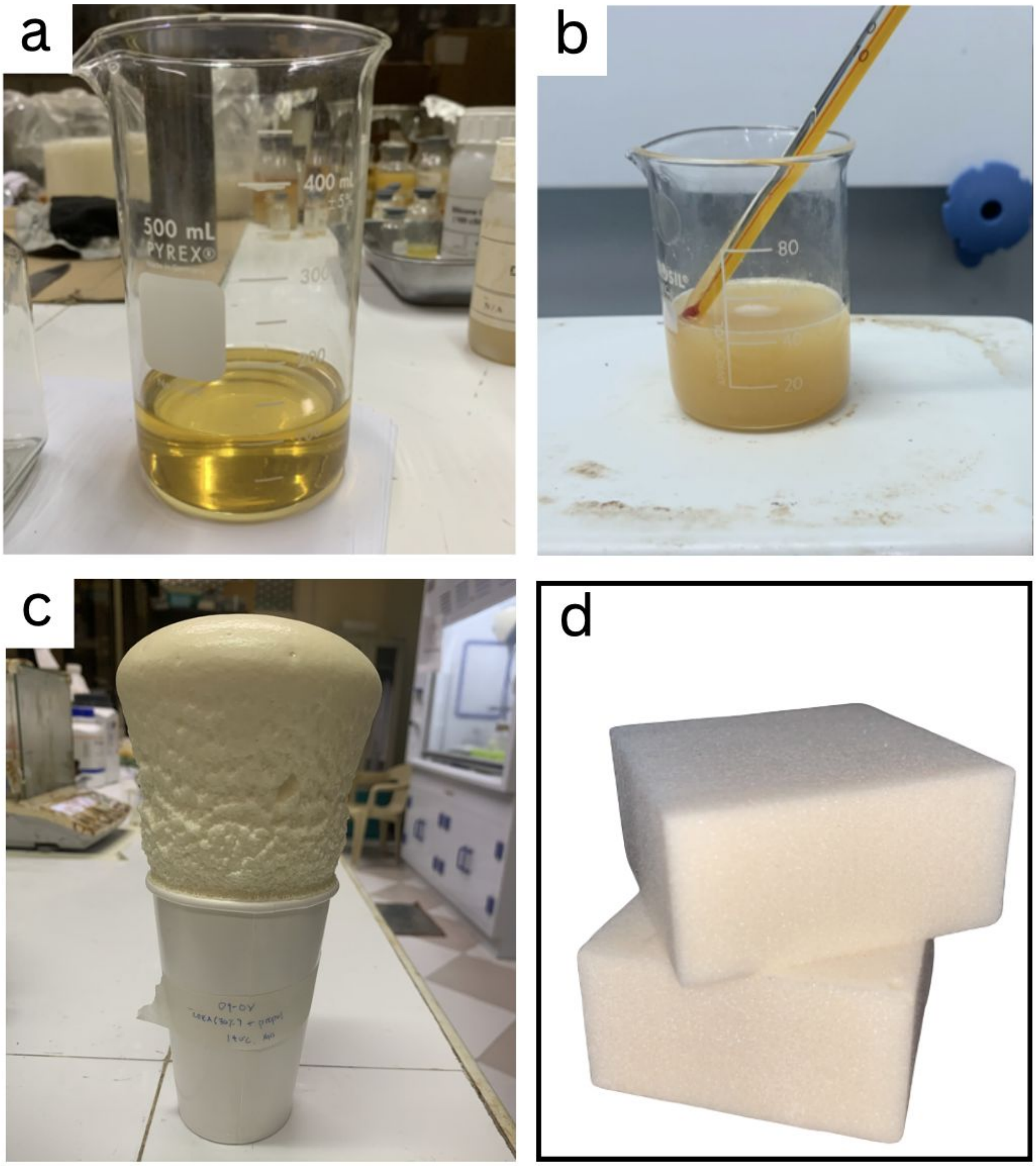

Fig. S1. Digital images of sequential amidation and prepolymerization process: (a) coconut diethanolamide (*p*-CDEA) product, (b) coconut oil polyurethane-urea prepolymer (COPUAP) synthesis, (c) COPUAP-based rigid polyurethane-urea foam (PU-COPUAP), (d) cured and cut (1x1 inch) PU-COPUAP in preparation for the compressive strength test

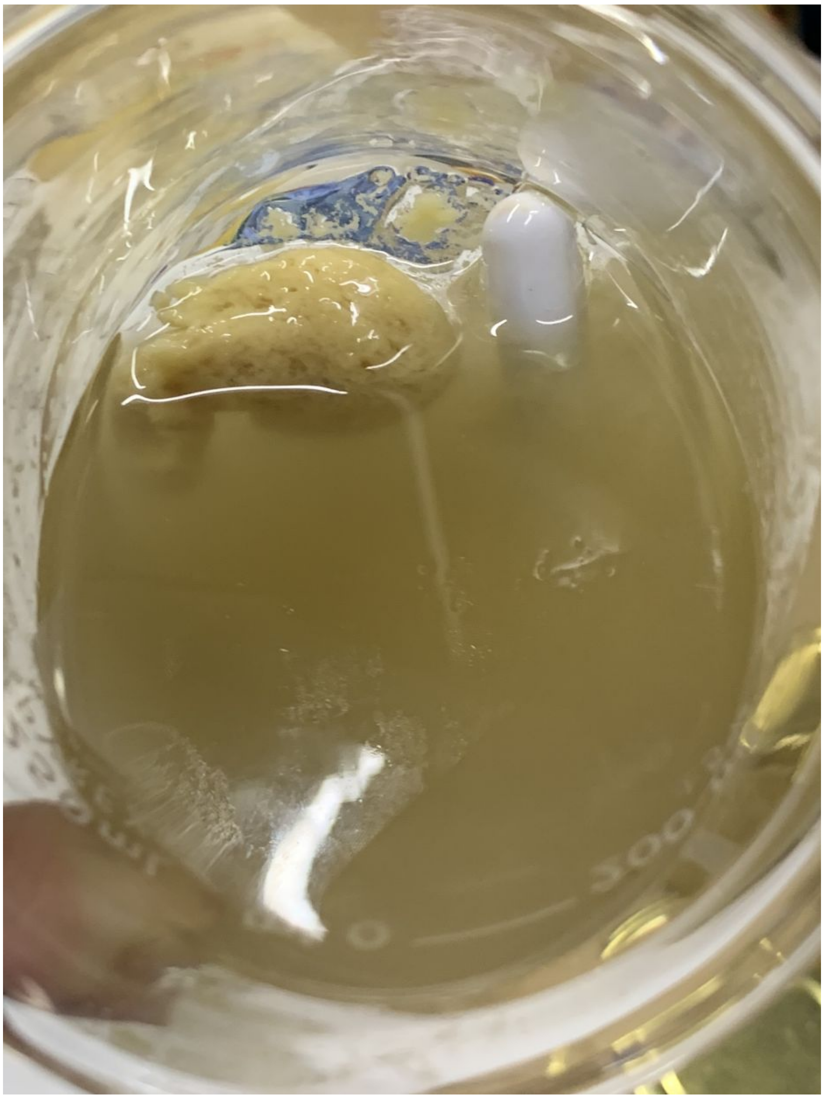

Fig. S2. Digital images depicting the formation of gelated pre-polymer material during prepolymerization after the MDI loading level exceeded 13%

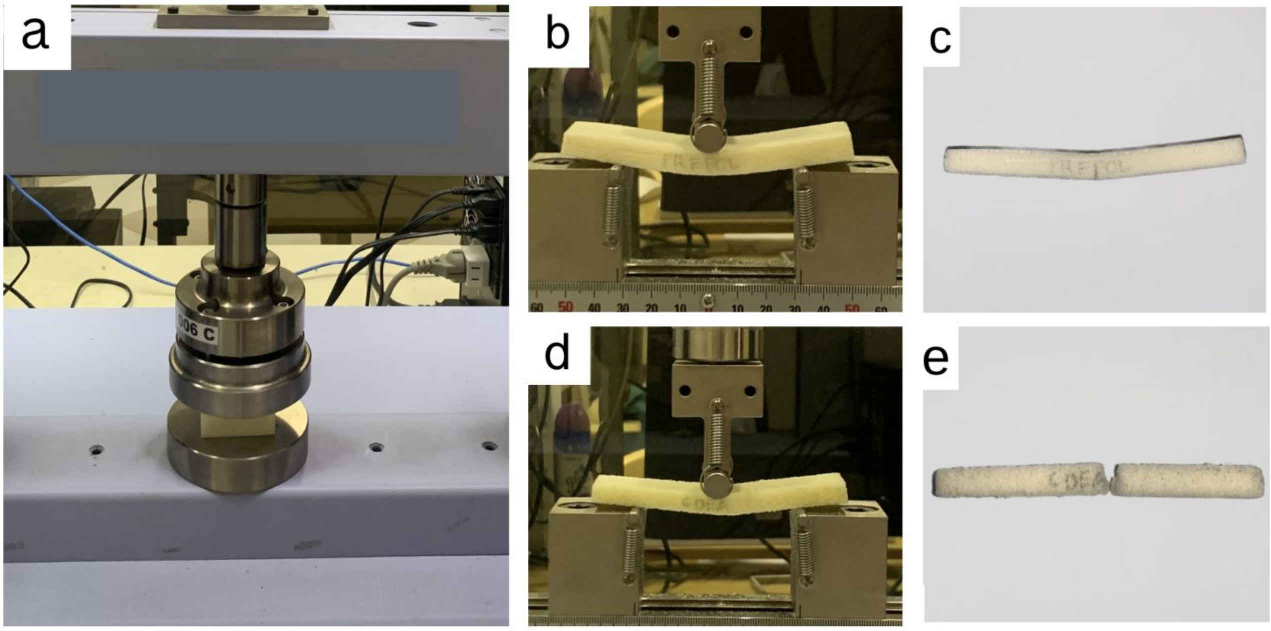

Fig. S3. Actual images (a) during compressive strength test of rigid polyurethane-urea foam (RPUAF), (b) during flexural test of coconut oil polyurethane-urea prepolymer-based rigid foam (PU-COPUAP), (c) PU-COPUAP after flexural test, (d) during flexural test of coconut diethanolamide-based rigid foam (PU-CDEA), and (e) PU-CDEA after flexural test

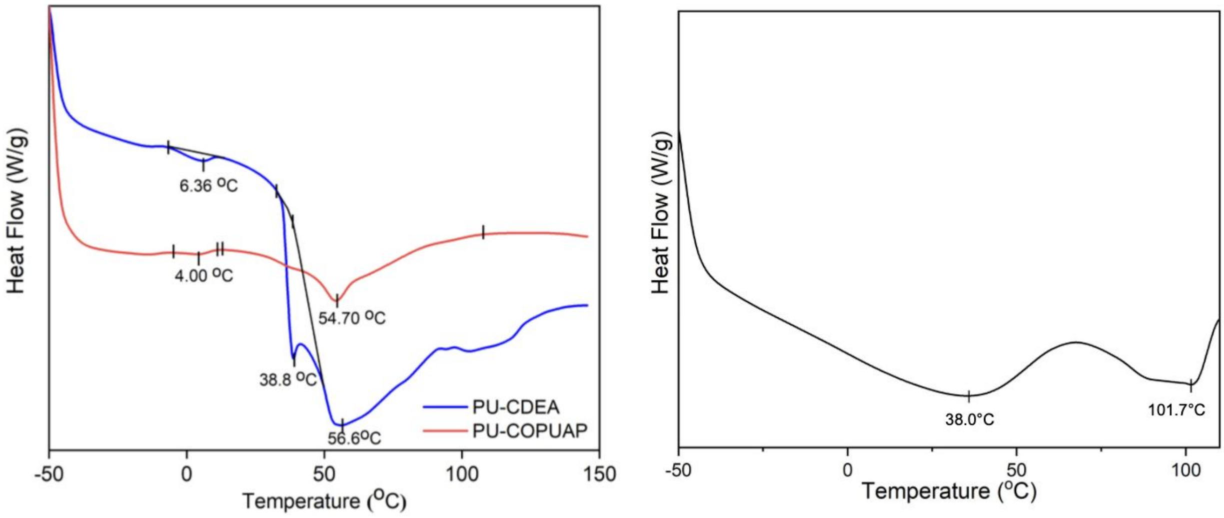

Fig. S4. Differential scanning calorimetry thermogram of (a) coconut diethanolamide-based rigid foam (PU-CDEA) and coconut oil polyurethane-urea prepolymer-based rigid foam (PU-COPUAP) and (b) MDI and DEA reaction showing thermal glass transition

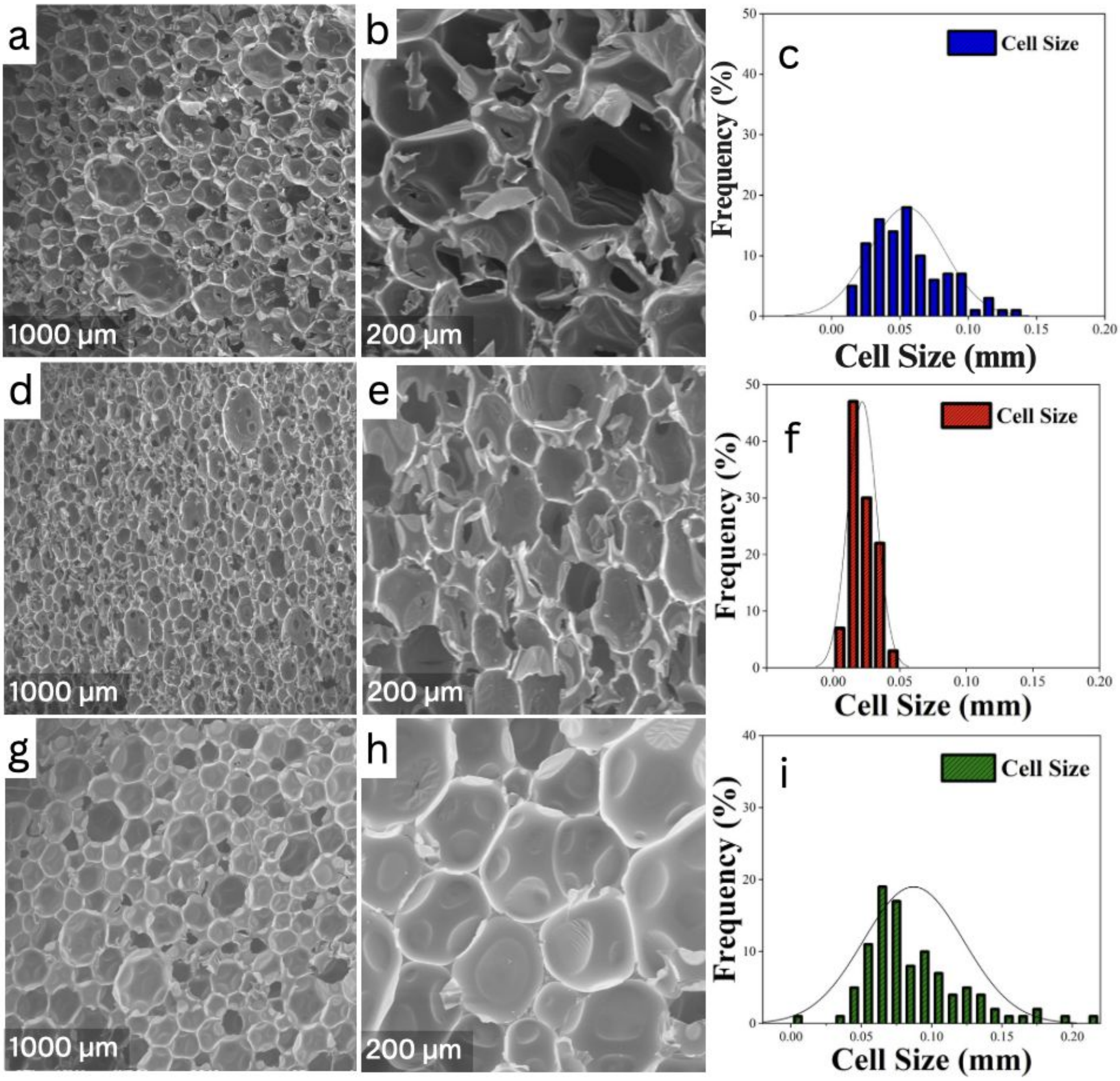

Fig. S5. SEM images of the external surface of (a-b) coconut diethanolamide-based rigid foam (PU-CDEA) 1000x magnification, (b) PU-CDEA 200x, (c) PU-CDEA cell size distribution, (d) coconut oil polyurethane-urea prepolymer-based rigid foam (PU-COPUAP) 1000x (e) PU- COPUAP 200x (f) PU-COPUAP cell size distribution (g) Voranol 490-based rigid foam (PU-V490) 1000x magnification, (h) PU-V490 200x, and (i) PU-V490 cell size distribution

References

[1] Campanella A, Bonnaillie LM, Wool RP. Polyurethane foams from Soyoil-based polyols. Journal of Applied Polymer Science. 2009;112(4):2567–78.

[2] Mohammed IA, Al-Mulla EA, Kadar NK, Ibrahim M. Structure-property studies of thermoplastic and thermosetting polyurethanes using palm and soya oils-based polyols. Journal of Oleo Science. 2013;62(12):1059–72.

[3] Zora N, Rigaux T, Buvat J-C, Lefebvre D, Leveneur S. Influence assessment of inlet parameters on thermal risk and productivity: Application to the epoxidation of vegetable oils. J Loss Prev Process Ind [Internet]. 2021;72(104551):104551. Available from: http://dx.doi.org/10.1016/j.jlp.2021.104551

[4] Borugadda VB, Goud VV. Hydroxylation and hexanoylation of epoxidized waste cooking oil and epoxidized waste cooking oil methyl esters: Process optimization and physico-chemical characterization. Ind Crops Prod [Internet]. 2019;133:151–9. Available from: http://dx.doi.org/10.1016/j.indcrop.2019.01.069

[5] Chaudhari AB, Tatiya PD, Hedaoo RK, Kulkarni RD, Gite VV. Polyurethane prepared from neem oil polyesteramides for self-healing anticorrosive coatings. Ind Eng Chem Res [Internet]. 2013;52(30):10189–97. Available from: http://dx.doi.org/10.1021/ie401237s

[6] Gobin M, Loulergue P, Audic J-L, Lemiègre L. Synthesis and characterisation of bio-based polyester materials from vegetable oil and short to long chain dicarboxylic acids. Industrial Crops and Products. 2015;70:213–20.

[7] Chaudhari A, Kuwar A, Mahulikar P, Hundiwale D, Kulkarni R, Gite V. Development of anticorrosive two pack polyurethane coatings based on modified fatty amide of Azadirachta indica Juss oil cured at room temperature – a sustainable resource. RSC Adv [Internet].

2014;4(34):17866–72. Available from: http://dx.doi.org/10.1039/c4ra01880j

[8] Standard Test Method for Apparent Density of Rigid Cellular Plastics. West Conshohocken, PA: ASTM International; 2003.

[9] Standard test method for compressive properties of rigid cellular plastics [Internet]. Astm.org. [cited 2023 Mar 13]. Available from: https://www.astm.org/d1621-04.html

[10] Standard test method for steady-state thermal transmission properties by means of the heat flow meter apparatus [Internet]. Astm.org. [cited 2023 Mar 13]. Available from: https://www.astm.org/standards/c518

[11] ISO 178:2019 [Internet]. ISO. 2019 [cited 2023 Mar 13]. Available from: https://www.iso.org/standard/70513.html

[12] Standard test methods for testing polyurethane raw materials determination of hydroxyl numbers of polyols [Internet]. Astm.org. [cited 2023 Mar 13]. Available from: https://www.astm.org/d4274-99.html
